# Lost and Found: Piwi and Argonaute Pathways in Flatworms

**DOI:** 10.3389/fcimb.2021.653695

**Published:** 2021-05-27

**Authors:** Santiago Fontenla, Gabriel Rinaldi, Jose F. Tort

**Affiliations:** ^1^ Departamento de Genética, Facultad de Medicina, Universidad de la República (UDELAR), Montevideo, Uruguay; ^2^ Wellcome Sanger Institute, Hinxton, United Kingdom

**Keywords:** Piwi, Ago, Vasa, RNAi pathways, miRNA, piRNA, siRNA, flatworms

## Abstract

Platyhelminthes comprise one of the major phyla of invertebrate animals, inhabiting a wide range of ecosystems, and one of the most successful in adapting to parasitic life. Small non-coding RNAs have been implicated in regulating complex developmental transitions in model parasitic species. Notably, parasitic flatworms have lost Piwi RNA pathways but gained a novel Argonaute gene. Herein, we analyzed, contrasted and compared the conservation of small RNA pathways among several free-living species (a paraphyletic group traditionally known as ‘turbellarians’) and parasitic species (organized in the monophyletic clade Neodermata) to disentangle possible adaptations during the transition to parasitism. Our findings showed that complete miRNA and RNAi pathways are present in all analyzed free-living flatworms. Remarkably, whilst all ‘turbellarians’ have Piwi proteins, these were lost in parasitic Neodermantans. Moreover, two clusters of Piwi class Argonaute genes are present in all ‘turbellarians’. Interestingly, we identified a divergent Piwi class Argonaute in free living flatworms exclusively, which we named ‘Fliwi’. In addition, other key proteins of the Piwi pathways were conserved in ‘turbellarians’, while none of them were detected in Neodermatans. Besides Piwi and the canonical Argonaute proteins, a flatworm-specific class of Argonautes (FL-Ago) was identified in the analyzed species confirming its ancestrallity to all Platyhelminthes. Remarkably, this clade was expanded in parasitic Neodermatans, but not in free-living species. These phyla-specific Argonautes showed lower sequence conservation compared to other Argonaute proteins, suggesting that they might have been subjected to high evolutionary rates. However, key residues involved in the interaction with the small RNA and mRNA cleavage in the canonical Argonautes were more conserved in the FL-Agos than in the Piwi Argonautes. Whether this is related to specialized functions and adaptations to parasitism in Neodermatans remains unclear. In conclusion, differences detected in gene conservation, sequence and structure of the Argonaute family suggest tentative biological and evolutionary diversifications that are unique to Platyhelminthes. The remarkable divergencies in the small RNA pathways between free-living and parasitic flatworms indicate that they may have been involved in the adaptation to parasitism of Neodermatans.

## Introduction

At the beginning of this century an unexpected and complex ‘RNA world’ started to be unveiled (Guil and Esteller, 2015), resulting in the discovery of novel layers of fine-tuned mechanisms for gene expression regulation, unimaginable until then. Regulatory activities were assigned to a growing range of new species of non-coding RNAs (Guil and Esteller, 2015). Single stranded non-coding RNAs of 20 to 30 nucleotides long are key mediators in small RNA pathways that underlie diverse biological processes. MicroRNAs (miRNAs) are post-transcriptional regulators involved in cell development and differentiation, metabolism, DNA methylation, neurological development, immune response, defense against viral infections and cancer (Huang and Zhang, 2014). Piwi-RNAs (piRNAs) are small non-coding RNAs specifically involved in the maintenance of genome stability by silencing transposable elements (TE) in germline cells (Weick and Miska, 2014). Finally, RNA interference (RNAi) is a pathway mediated by short-interfering RNAs (siRNAs) that might have originally emerged as response to double-strand RNA (dsRNA) generated during some virus infections (Ding and Voinnet, 2007). The presence of dsRNA molecules in the cell cytoplasm triggers a post-transcriptional degradation of complementary mRNA molecules. Consequently, this pathway has been exploited as a reverse genetic tool to silence specific genes (Han, 2018). Currently, more than two decades after it was first applied to a flatworm species (Sánchez Alvarado and Newmark, 1999), it is still the main tool to study gene function in worms (Mourão et al., 2012; Wang et al., 2020).

The regulatory pathways mediated by small RNAs have been extensively studied in the nematode *Caenorhabditis elegans*, first model species in which post-transcriptional gene-silencing mediated by dsRNA was described (Fire et al., 1998). In addition, *C. elegans* was the first organism from which a miRNA was isolated: lin-4, (Lee, 1993). On the other hand, *Schmidtea mediterranea* was the first free-living flatworm species to be silenced by RNAi (Sánchez Alvarado and Newmark, 1999). Planarians have long been models for tissue regeneration and stem cells homeostasis, and the emergence of RNAi as functional genomic tool has transformed the field (Reddien and Alvarado, 2004; Blythe et al., 2010; Sandmann et al., 2011).

Planarians are free living flatworms of the order Tricladida, phylum Platyhelminthes. Platyhelminthes are one of the major phyla of invertebrate animals, traditionally divided into four classes: the free living ‘turbellarians’, the ectoparasitic Monogenea, and the endoparasitic Trematoda (flukes) and Cestoda (tapeworms). All the parasitic classes are grouped in Neodermata given they all share the presence of a syncytial unciliated epidermis (the neodermis) that seem to be crucial for host immune system evasion and nutrient absorption (Caira and Littlewood, 2013). Studies based on rRNA (Larsson and Jondelius, 2008; Laumer and Giribet, 2014) and transcriptomic data (Egger et al., 2015; Laumer et al., 2015) showed that the ‘turbellarians’ constitute a paraphyletic group, splitting now the phylum Platyhelminthes into two clades; the ancestral Catenulida and the Rhabditophora, that contains several free-living orders and the parasitic neodermatans. More recently the Macrostomorpha was placed as the earliest diverging Rhabditophoran linage and the Tricladida as part of the later evolved ‘turbellarians’ (Egger et al., 2015; Laumer et al., 2015) (**Figure S1**).

The success of RNAi in planarians encouraged its use in parasitic species where genetic tools were desperately needed. RNAi has proven to be functional in other free-living and parasitic species (Orii et al., 2003; Rinaldi et al., 2008; Kuales et al., 2011; Dell’Oca et al., 2014; Moguel et al., 2015) and miRNAs have been detected in almost all flatworm lineages (Palakodeti et al., 2006; Cucher et al., 2011; Fromm et al., 2013; Fontenla et al., 2015; Cai et al., 2016; Protasio et al., 2017). Whilst piRNAs were early found in free living planarians (Palakodeti et al., 2008; Friedländer et al., 2009), remarkably, they have not been identified in parasitic species.

Our previous analysis of the small RNA pathways in parasitic flatworm genomes strongly indicated relevant gene losses within the neodermatans that may have been associated with the adaptation to parasitism (Fontenla et al., 2017). However, these observations were limited by the paucity of data from free-living species, represented only by *Macrostomum lignano* and the planaria *S. mediterranea.*

The availability of transcriptomic data from several early diverging free-living species (Laumer et al., 2015) allowed us now to expand our analysis and provide a complete picture of the phylum Platyhelminthes, including a representative set of ‘turbellarian’ species^1^, adding also novel monogenean (Ilgová et al., 2017) and trematode genomes (Oey et al., 2018; Choi et al., 2020; Rosa et al., 2020). The emerging picture provides evidence of substantial differences in the distribution of small RNA pathways proteins suggestive of diverse regulatory possibilities in both free living and parasitic flatworms. Additionally, these findings shine a light into tentative relations between the divergency of small RNA pathways and mechanisms driving parasitism in organisms that are responsible for an enormous disease burden in both human and animals.

## Methods

### Data Acquisition

Small RNA pathways proteins of *Macrostomum lignano*, *Schmidtea mediterranea*, *Gyrodactylus salaris, Schistosoma mansoni* and *Echinococcus multilocularis* together with other Neodermata species were characterized as described (Fontenla et al., 2017). The recently published genomes of the trematodes *Fasciola gigantica* and *Fasciolopsis buski* (Choi et al., 2020) and four species of the genus *Paragonimus* (Oey et al., 2018; Rosa et al., 2020) were also included. Transcriptomic data on several early diverging flatworms (Laumer et al., 2015) were obtained from public repository Data Dryad (doi: 10.5061/dryad.622q4). Transcriptomic data on *Eudiplozoon nipponicum* was downloaded from GitHub repository (Ilgová et al., 2017). To study the quality of the transcriptomic data, BUSCO v4.1.4 (Seppey et al., 2019) was used with option –l metazoa to search for conserved Metazoan genes. Considering the levels of missing and fragmented transcripts we selected the seven best ‘turbellarian’ assemblies, comprising a reasonable overview of the ‘turbellarian’ clade diversity. The species analyzed were *Stenostomum leucops*, *Prostheceraeus vittatus*, *Geocentrophora applanata*, *Rhynchomesostoma rostratum*, *Monocelis fusca*, *Kronborgia cf. amphipodicola* and *Bothrioplana semperi*. TransDecoder.LongOrfs function of TransDecoder v4.1.0 software (available at https://github.com/TransDecoder/TransDecoder/) was used to predict longest open reading frames (ORFs) on transcripts. Detailed information and source of other sequences used in the construction of the gene trees can be found in **Table S10**.

### Identification of Small RNA Pathways Proteins

Flatworm small RNA pathways proteins previously identified by us (Fontenla et al., 2017), and *C. elegans* factors that we failed to detect in our previous search were used as query to interrogate with BLASTp the translated transcriptomes and genomes. We also inspected the presence of *D. melanogaster*’s Zuc and Vasa (Fontenla et al., 2017) using the same approach against all the species. Matched sequences were acquired and used to perform reverse BLASTp against the proteomes of *S. mansoni*, *M. lignano*, *C. elegans* and *D. melanogaster* retaining only the best hit. HMMScan (Johnson et al., 2010) was used to predict the functional domains in putative small RNA pathways proteins, and sequences with no functional domains were discarded from the analysis. HMMScan prediction was performed in the complete transcriptomes of *S. mediterranea* and *S. mansoni* as quality control, to confirm that distant homologous genes with the conserved function were not discarded in the BLAST search. The procedure did not show a different outcome to the blast results Detected sequences are available as **Supplementary Material** (**Folder S1**).

### Construction of Phylogenetic Trees

**Figure 1:**
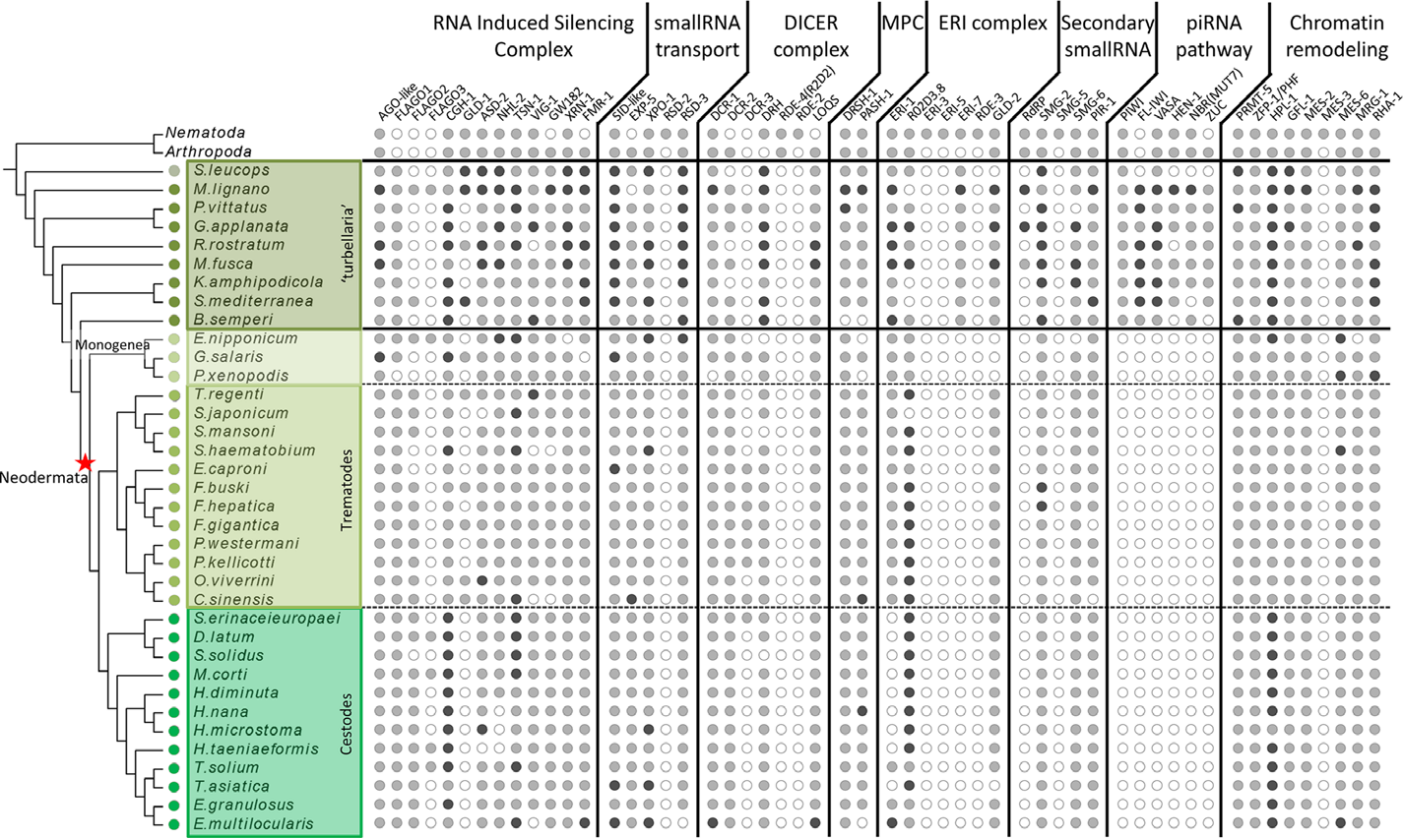
Distribution of miRNA, piRNA and RNAi proteins across flatworms, arthropods and nematodes. White circles indicate absence, grey circles indicate one homologue detected, black circles indicate two or more homologues detected. In arthropods and nematodes only presence/absence is indicated.

To avoid overestimating the number of genes in transcriptomic data or report entire genome duplications as gene expansions, CD-HIT (Huang et al., 2010) was used to cluster sequences with more than 90% similarity (option –c 0.9). MAFFT (Katoh and Standley, 2013) with local alignment option and structural information was used to align the selected sequences. Due to the fragmented nature of transcriptomic data, sequence alignments were manually curated using BioEdit (Hall, 1999), removing sequences that were too short (< 140 aa). Maximum Likelihood trees with statistical branch support (SH-like) were generated with PhyML (Guindon et al., 2010), with models inferred with Smart Model Selection (SMS) (Lefort et al., 2017). Trees were visualized with Evolview (He et al., 2016), and enriched by adding domain structure information. Argonaute unrooted tree (**Figure 1**) was visualized with MEGA version X (Kumar et al., 2018).

### Molecular Evolutionary Analysis of Argonaute Subfamilies

For the heatmap showing the sequence conservation of the Argonaute subfamilies, the consensus function of R package seqinR (Charif and Lobry, 2007) was used to build matrices with the residues count at each position of the alignments. Next, we applied a color scale to the most abundant residue at each position. To calculate the percentage of conserved positions by domain, the positions with conserved residues in more than 50% of the sequences were added and corrected by the domain length.

For positive selection inference, Argonaute transcripts were codon aligned in MEGA version X (Kumar et al., 2018) with Muscle aligner. The alignment was edited with BioEdit (Hall, 1999) and MEGA version X Codon-based Z-test of Selection tool used to compute synonymous and nonsynonymous substitutions: the hypothesis tested was positive selection (dN>dS) in sequence pairs with the Nei-Gojobori method (Nei and Gojobori, 1986), with a *p-value* threshold ≤ 0.05.

Additionally, we tested for evidence of positive selected sites (PSSs) using the mixed effects model of evolution (MEME) (Murrell et al., 2012) method. MEME applies a branch-site random effects phylogenetic framework that allows the distribution of dN/dS to vary from site to site as well as from branch to branch, thereby identifying residues that have undergone episodic selection (i.e. positive selection that varies temporally throughout the tree). Only likelihood ratio test (LRT) with *p-value* ≤ 0.05 were considered as statistically significant evidence of PSSs.

## Results

### While miRNA and siRNA Pathways Are Conserved Across Platyhelminths, the Complete Piwi Pathway Is Lost in Parasitic Flatworms

The presence of small RNA pathways was investigated on available transcriptomes from 25 early diverging flatworms (Laumer et al., 2015). We first evaluated the quality of the assemblies against a set of conserved metazoans genes using BUSCO v 4.1.4. Based on the level of fragmentation and number of missing orthologues, we selected assemblies from seven species, that added to *M. lignano*, and *S. mediterranea* capture the diversity of free-living flatworms. In addition, we included a novel dataset from underrepresented monogeneans and six novel trematode genomes (Oey et al., 2018; Choi et al., 2020; Rosa et al., 2020). Taken together this dataset provides a comprehensive phylogenetic view of the platyhelminth clade diversity (**Figure S1**).

The homology search with a curated set of proteins involved in small RNA pathways not only showed the presence of most of them in all free-living species, but also outstanding absences in neodermatans. While several proteins show differential distribution among diverse classes (**Figures S2**–**S6** and **Tables S1**–**S6**), a remarkable feature is the complete absence of all the piwi pathway components in all parasitic species (**Figure 1**).

The absence of piwi proteins in parasitic trematodes and cestodes have been previously proposed (Skinner et al., 2014; Fontenla et al., 2017), raising questions on how the parasitic species control the activity of repetitive mobile elements. Our extensive search of other piwi pathway genes across flatworms clearly shows that the complete pathway is missing in neodermatans while is conserved in free-living species.

### Amplifications in the Argonaute Family Show Differential Distributions Across Flatworms

Argonautes, small-RNA binding proteins, are central components of all the small RNA pathways (Niaz, 2018). Phylogenetic studies have traditionally classified the family into the Ago class (that is further subdivided in two subclasses comprising miRNA Agos and the siRNA associated proteins), the Piwi class, and the Wago clade, this latter, comprising nematode specific argonaute proteins (Wynant et al., 2017).

The comparison of Argonaute superfamily proteins from diverse metazoans, now including our extended sampling of flatworms, reveals interesting differences in their distribution, particularly between free-living and parasitic species.

All flatworms have putative orthologues to miRNA class proteins that constitute a well-defined clade.

**Figure 2:**
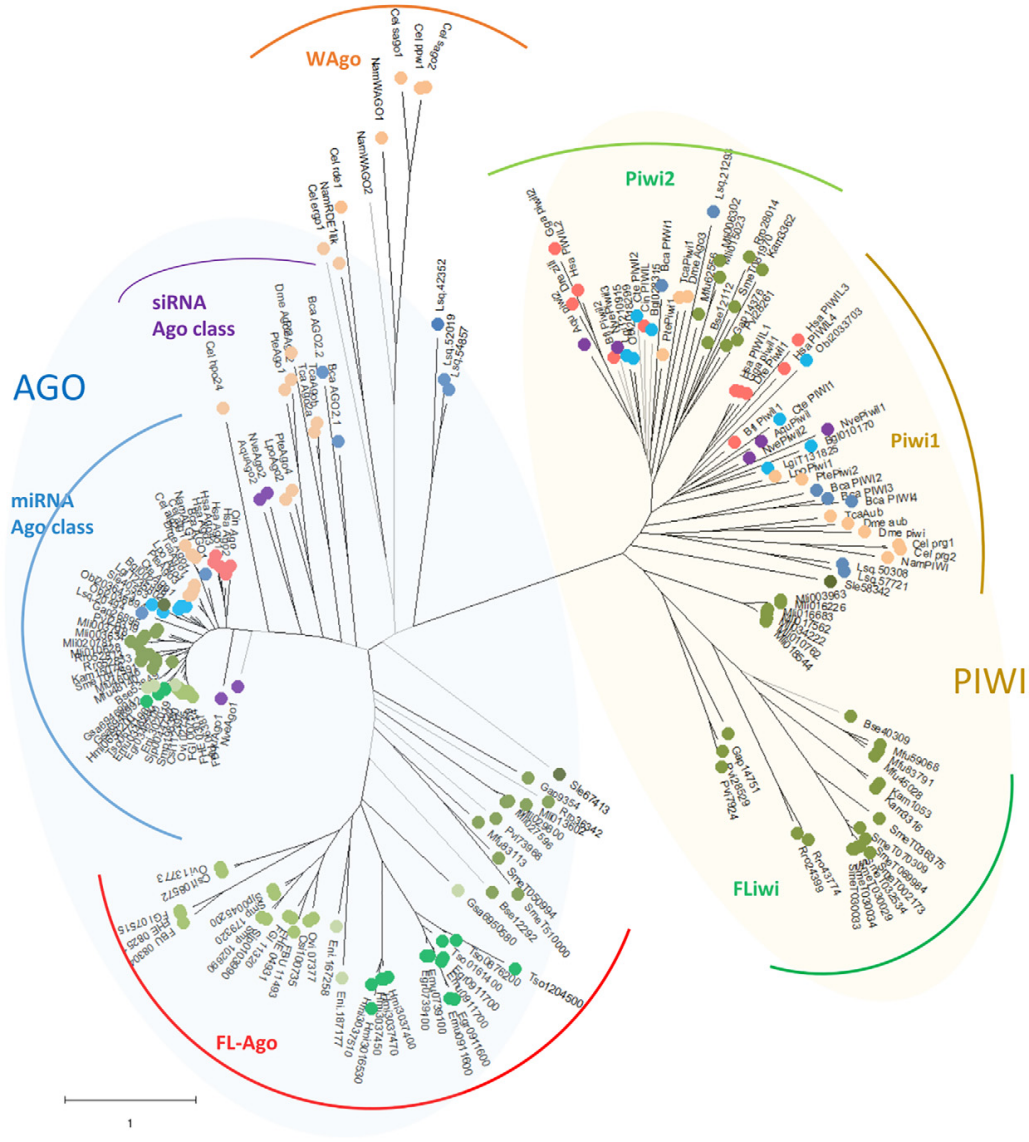
Unrooted maximum likelihood phylogenetic tree of Argonaute proteins belonging to Deuterostomia (•: Hsa, *Homo sapiens*; Gga, *Gallus gallus*; Dre, *Danio renio*; Bfl, *Branchiostoma floridae*; Cin, *Ciona intestinalis*), Ecdysozoa (•: Cel, *Caenorhabditis elegans*; Nam, *Necator americanus*; Dme, *Drosophila melanogaster*; Tca, *Tribolium casteneum*; Pte, *Parasteatoda tepidariorum*; Lpo, *Limulus polyphemus*), Mollusca & Annelida (•: Obi, *Octopus bimaculoides*; Bgl, *Biomphalaria glabrata*; Lgi, *Lottia gigantea*; Cte, *Capitella teleta*), Rotifera & Gastrotricha (•: Bca, *Brachionus calyciflorus*; Lsq, *Lepidermella squamata*) ‘turbellaria’ Catenulida (•: Sle, *Stenostomum leucops*), ‘turbellaria’ Rhabditophora (•: Mli, *Macrostomum lignano*; Pvi, *Prostheceraeus vittatus*; Gap, *Geocentrophora applanate*; Rro, *Rhynchomesostoma rostratum*; Mfu, *Monocelis fusca*; Kam, *Kronborgia amphipodicola*; Bse, *Bothrioplana semperi*), Monogenea (•: Eni, *Eudiplozoon nipponicum*; Gsa, *Gyrodactilus salaris*), Trematode (•: FBU, *Fasciolopsis buski*; FHE, *Fasciola hepatica*; FGI, *Fasciola gigantica*; Ovi, *Opisthorchis viverrini*; *Clonorchis sinensis*; Smp, *Schistosoma mansoni*; Sjp, *Schistosoma japonicum*), Cestode (•: Hmi, *Hymenolepis microstoma*; Tso, *Taenia solium*; Emu, *Echinococcus multilocularis*; Egr, *Echinococcus granulosus*), Cnidaria & Porifera (•: Nve, *Nemastella vectensis*; Aqu, *Amphimedon queenslandica*). Black branches indicate SH-like approximate likelihood ratios ≥ 90. Arches indicates the subgroups of Argonautes (blue shaded) and Piwi (green shaded) genes.

Rather than grouping with siRNA class Agos from basal metazoans (poriferans and cnidarians), ecdysozoans (nematodes and arthropods) or other lophotrochozoa (mollusks, annelids, gastrotrichs and rotifers), all the other flatworm Ago sequences cluster together in a well-defined clade (**Figure 2**). This flatworm specific clade has been previously reported by us and others, and termed FLAgos (Zheng, 2012; Skinner et al., 2014; Fontenla et al., 2017).

**Figure 3:**
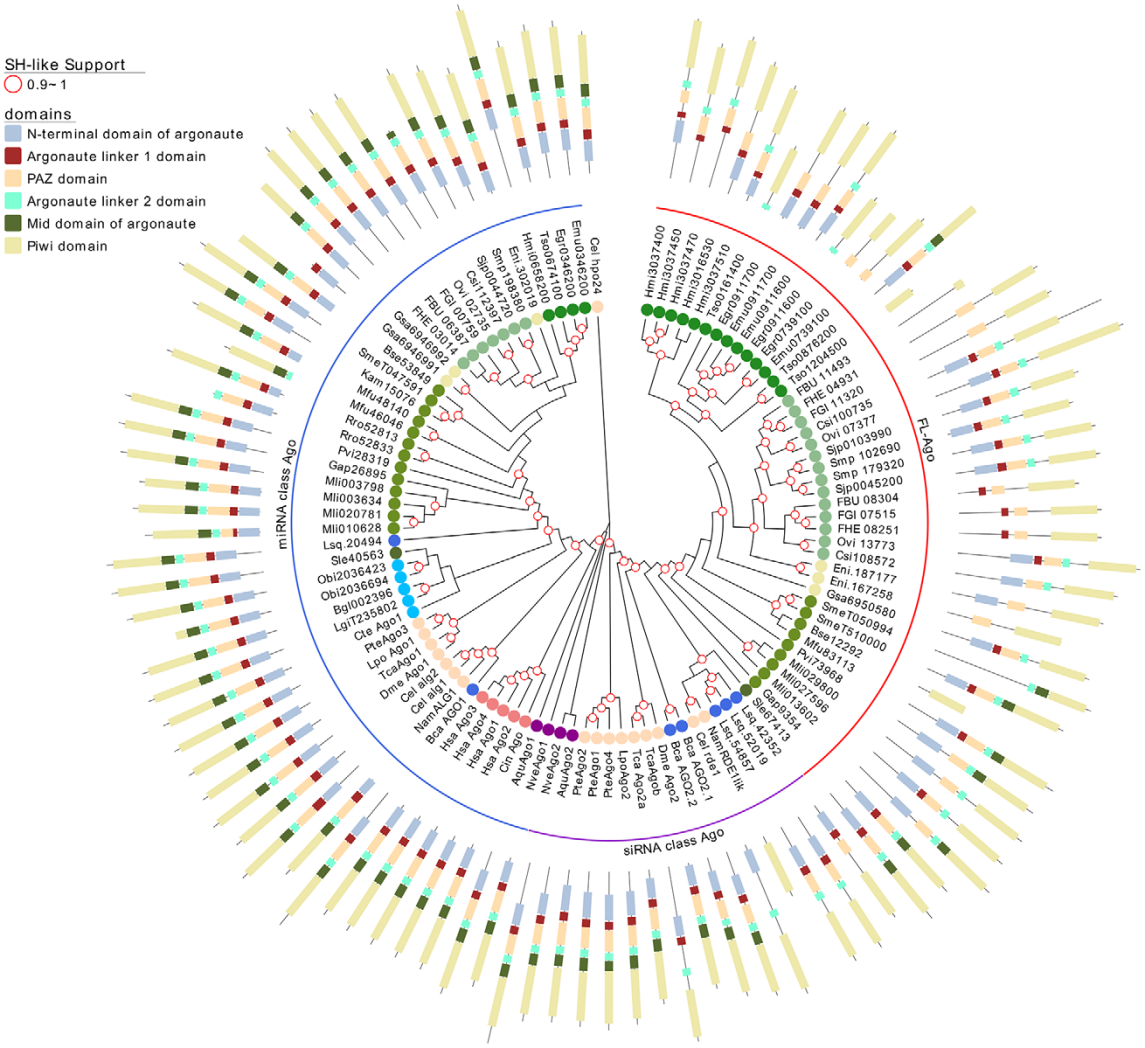
A maximum likelihood tree of canonical Argonaute proteins and FLAgos. SH-like approximate likelihood ratios are indicated. Conserved protein domains were predicted with HMMScan. Blue, purple and red arches correspond to miRNA Ago-class, siRNA Ago-class and FL-Agos, respectively. Abbreviations are as indicated in **Figure 2**.

Interestingly, while a single gene is found in all the free-living species (with the only exception of *S. mediterranea* that showed a gene duplication), the parasitic cestodes and trematodes have experienced gene amplifications leading to two or more genes (**Figures 2** and **3**). The two FLAgo genes from the model trematode *S. mansoni* (Smp_179320 and Smp_102690) are organized in tandem in chromosome 1, but show clear differential expression among developmental stages (**Figure S7**). While a similar tandem gene arrangement could be detected in *S. japonicum* genome, it is not possible to assess if this is a general trend in trematodes due to the still fragmentary nature of the assemblies for other species. Similarly, within cestodes three FLAgo genes are in tandem in *Echinococcus multilocularis* and a more complex array of amplified genes is evident in *Hymenolepis microstoma*. With the information available so far these amplifications appear as independent events in cestodes and trematodes (**Figure 3**). Since single genes are recovered in all free-living species, a more parsimonious hypothesis would be an initial duplication at the origin of neodermatans. However, more detailed analyses and better genome assemblies are needed, in particular considering that the gene trees suggest these are rapidly evolving Agos.

**Figure 4:**
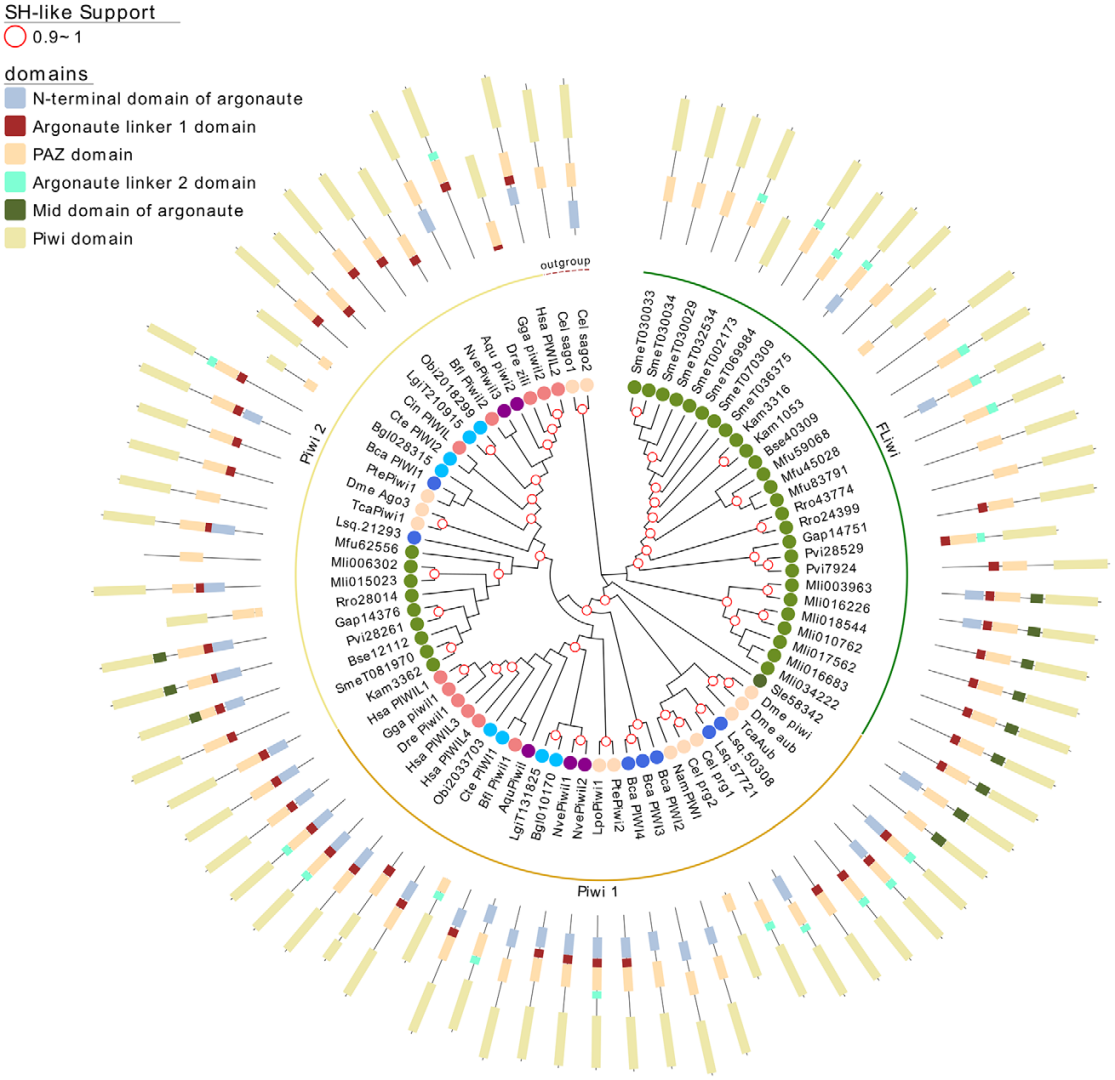
A maximum likelihood tree of Piwi proteins. Wagos were used as outgroup. SH-like approximate likelihood ratios are indicated. Conserved protein domains were predicted with HMMScan. Light green, yellow and green arches corresponds to the cluster of the Piwi 2, Piwi 1 and FLiwis, respectively. Abbreviations are as indicated in **Figure 2**.

Two subgroups of piwi class genes have been previously described (Wynant et al., 2017; Jehn et al., 2018). Consistently within the free-living flatworms Piwis two sub-groups are evident. But while single genes from all free-living species cluster within the Piwi2 clade, a second separate clade is formed with all the remaining Piwi genes from free-living Rhabditophorans, here termed FLiwi (**Figures 2** and **4**). The complete absence of any piwi homologue in neodermantans is quite evident in monogeneans, trematodes and cestodes, and this is confirmed based on the extensive sampling on available genomes.

The FLiwi cluster showed several independent gene duplications that have probably occurred after speciation, although we cannot rule out that gene duplications might be overestimated in those species in those species for which only transcriptomic data is available. We cannot rule out that transcripts from different genes were counted as one if the sequences were too similar due to very recent gene duplication events, as was the case for *S. mediterranea*’s FLiwis (Kim et al., 2020). Interestingly, *S. mediterranea* displayed the widest expansion of FLiwis (**Figure 4** dark green arch). Here, eight genes clustered in this group, some with identical sequences that can be collapsed to five quite similar genes (SmeT032534, SmeT030029, SmeT030034 and SmeT030033 present >99% identity). Whether these are functional genes or represent transcribed pseudogenes remains unknown (Kim et al., 2020).

Interestingly, single Ago (Sle67413) and Piwi (Sle58342) genes can be detected in the catenulid *S. leucops*, that generally is placed outside of the clades from other flatworms. It is not possible to assess if the absence of further genes is real or is due to partial sampling of the available transcriptome. In any case, it is quite interesting the placing outside of other flatworms since the inclusion of the group within platyhelminthes was in debate until recently (Larsson and Jondelius, 2008; Laumer and Giribet, 2014).

The phylogenetic tree shown in **Figure 2** suggests that FLAgo and FLiwi have probably experimented highly evolutionary rates, as indicated by their long branches. This may also explain the observed branching pattern inconsistent with the species tree (**Figure 2**). In this sense, a parsimonious explanation would be that FLAgo might represent rapidly evolving siRNA class genes, while Fliwi could correspond to fast evolving piwi1 type proteins. Further evidence is needed to functionally validate these proteins.; therefore, we decided to investigate other aspects of these intriguing genes.

### FLAgos and FLiwis Structures Are More Variable Than Canonical Ago and Piwi Counterparts

Argonaute proteins consist of five distinct domains: the N-terminal, PAZ, Mid, PIWI and two linker regions, L1 and L2. When analyzing the domain conservation among the Argonaute proteins detected across flatworms, it was obvious that while canonical miAgo class genes are highly conserved in their structure, FL-Agos have a more variable structure with MID domain being poorly detected. Both Piwi subfamilies display PAZ and PIWI domains, and while the N-terminal and linker 1 domain are generally identified in the free-living Piwi 2 genes, they are devoid of the linker 2 domain (**Figures 3** and **4**). Conversely, although more structurally variable probably due to independent gene amplifications, most of the FLiwi proteins have linker 2 domain in addition to PAZ/PIWI domains, while the detection of the other domains is more scattered.

**Figure 5:**
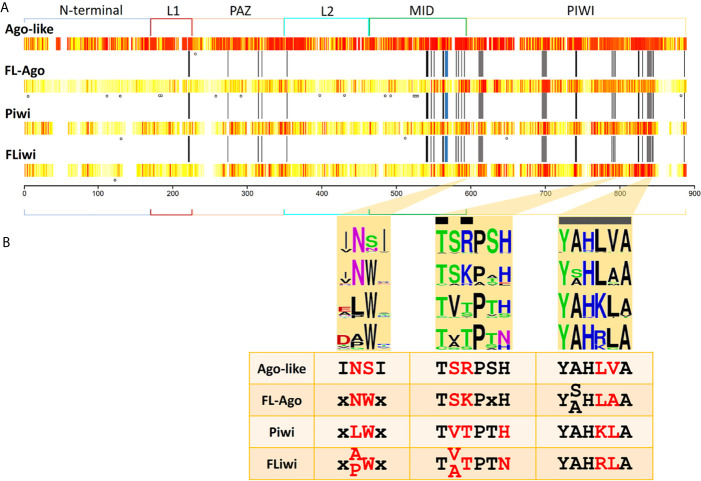
Sequence conservation among Argonaute subfamilies. **(A)** Conservation is shown in a white (less conserved) to red (most conserved) color scale. N-terminal, linker 1 (L1), PAZ, linker 2 (L2), MID and PIWI domains are indicated. Black bars indicate the positions that have been reported to interact with the miRNA (Elkayam et al., 2012). Grey bars indicate the positions of the catalytic DDH triad. Blue bar indicate the position that has been reported to interact with the seed region of miRNAs (Maldonado et al., 2017). Dots indicate positions with statistically significant LRT (p-value ≤ 0.05) detected with MEME tool. **(B)** Highlight of three regions of the MID and PIWI domains that produce a signature for each Ago subfamily. Black bars indicate the positions that have been reported to interact with the miRNA. Grey bars indicate the positions of the catalytic DDH triad.

Next, we studied the conservation at sequence level of the flatworm Argonaute genes. The canonical miAgo family showed the highest overall conservation which reached up to 85% of conserved positions in the MID domain (**Figure 5A** and **Table S7** for complete list of conservation by domain). Piwi, FLiwi and FLAgos showed lower general sequence conservation. The most conserved domain across clades at amino acid level is the PIWI domain (**Figure 5A**). Remarkably, the less conserved domain in the canonical Ago (i.e. the N-terminal domain) showed more conservation than any of the domains in both Piwi clades and FL-Agos (**Figure 5A** and **Table S7**). This is suggestive of faster evolution rates of the FLAgo and FLiwi clades, consistent with previous reports (Wynant et al., 2017).

Since the differences in sequences conservation may be associated with selection, we measured the variation in the rate of non-synonymous/synonymous (dN/dS) substitutions of Argonaute family proteins. We found that within each subgroup of Argonautes, the FLAgos showed the highest number of statistically significant dN/dS substitutions when free-living to parasitic pairs of genes were considered (**Table S8**). The second group with dN>dS was the FLiwis followed by the miRNA class Agos and the Piwis. When considering variation between subfamilies the highest rate of dN/dS substitutions were observed between the Agos *vs* FLAgos, Ago/Piwis, followed by Piwi/FLAgos and Ago/FLiwi. Using MEME, we aimed to detect sites that were subjected to positive or diversifying selection (see *Methods*). FLAgos showed the highest count of positions under positive diversifying selection, with a significant likelihood ratio test (LRT) of non-synonymous to synonymous substitutions (p-value ≤ 0.05) (dots in **Figure 5A**). Meanwhile, the Piwis showed 3 putative sites under positive selection, and the FLiwis and canonical Agos groups only had one position each (**Figure 5A**).

### Small RNA Binding and Catalytic Amino Acids Conservation in FLAgos and FLiwis

Structural studies have shined a light on the mechanisms of action of Argonautes. Human Ago2 bonded to miRNA has a bi-lobed architecture, with the guide miRNA threaded through a central cleft formed by the N-PAZ and MID-PIWI domains. Multitude interactions within this tight binding pocket were identified, involving mainly residues of the MID domain capped on the 5’ side by PIWI domain residues (Elkayam et al., 2012; Kong et al., 2017). There, the target mRNA has access to mate with the miRNA and is cleaved by a RNAase H fold comprised by the Asp-Asp-His (DDH) triad in the PIWI domain (Tolia and Joshua-Tor, 2007). Consequently, we sought conservation of these active sites and functional residues in the different Ago classes (indicated by black and grey bars at **Figure 5A**).

Fourteen out of 51 functionally relevant positions were generally conserved in all the Argonaute proteins. Further fifteen positions were conserved between Ago-like and FLAgos, and a minor group of residues were shared between Piwi and FLiwi (**Figure S8**). These include four of the eight residues involved in positioning the guide RNA with respect to the active site to ensure that cleavage of targets occurs at a well-defined and predictable position. Three other positions are conserved only between canonical Agos and FLAgos (**Figure S8**). On the other hand, both Piwis and FLiwis present well conserved substitutions at positions 588 [K→ polar (Q)] and 845 [R→ hydrophobic (L, M or F)] (**Figure S8**), that could also be relevant in the binding to the small RNA.

The QSKN motif (positions 566 to 569) of the MID domain (blue bar in **Figures 5A** and **S8**) was reported to be involved in the binding to the seed region of the miRNA in the *Echinococcus canadensis* canonical Ago genes (Maldonado et al., 2017). Interestingly, while this QSKN motif is conserved in all Neodermatans, (**Figure S8**), the second position was occupied by a non-polar Alanine residue (QAKN), in ‘turbellarians’ as in *D. melanogaster* and *C. elegans.* In Deuterostomes, the same position was occupied by the non-polar aliphatic residue Valine (QVKN) (or Methionine in Has_Ago2, QMKN). Although the functional implications of this substitution are not clear, the restricted conservation of this motif in the Ago-like genes suggests that it is relevant in this subfamily, and importantly, may be useful as a marker of linage in the future.

The RNase H activity associated with a conserved DDH triad (grey bars in **Figures 5A** and **S8**) is well conserved in all the miRNA class Ago genes of Platyhelminthes. In the case of the Piwi class subfamily only SmeT002173 possess substitutions in the catalytic triad. The absence of the DDH triad would not imply that the catalytic activity was lost, as Dme_piwi has been shown to possess “slicer” activity even though it contains a DDK active site (Tolia and Joshua-Tor, 2007). Furthermore, it was shown for Hsa Ago3 that irrespective of RNase triad conservation, the catalytic activity changes depending on the guide RNA that is loaded (Park et al., 2017). In any event, additional *in silico* approaches as structural homology modeling, ligand docking and molecular dynamics, as well as, experimental evidence involving site-specific mutagenesis and cleavage assays are needed to define the ligand-protein interaction and characterize the catalytic activity of the diverse flatworm Argonautes.

Based on the residue conservation we selected three short motifs with different conserved residues among the diverse flatworm Argonaute subfamilies that can be useful to differentiate them, classifying and assigning novel members (**Figure 5B**). The first motif consists of a duo located in the carboxi-terminal end of the MID domain and is an Asn (N) preferentially followed by Ser (S) in canonical Agos and Asn-Trp (NW) in FLAgos. The non-polar Asn is substituted by Leu (L) and Ala (A) or Pro (P) in Piwi and FLiwis, respectively. The second and third motifs are within the PIWI domain. Motif 2 is TSRPSH in miRNA class Agos, while is TSKPxH in FLAgos, TVTPTH in Piwi and TVTPTN or TATPTN in FLiwis. The third motif contains the Histidine residue of the DDH triad and corresponds to a sextet that has the sequence YALHVA in Ago-like, YSLHAA or YALHAA in FLAgos, YAHKLA in Piwis or YAHRLA in FLiwis. We suggest that the analysis of these motifs may be useful to classify Argonaute proteins in flatworms, and also might provide a means of rapidly identify members in other metazoan species.

### Trematodes Display a Shorter Dicer-2 Gene

**Figure 6:**
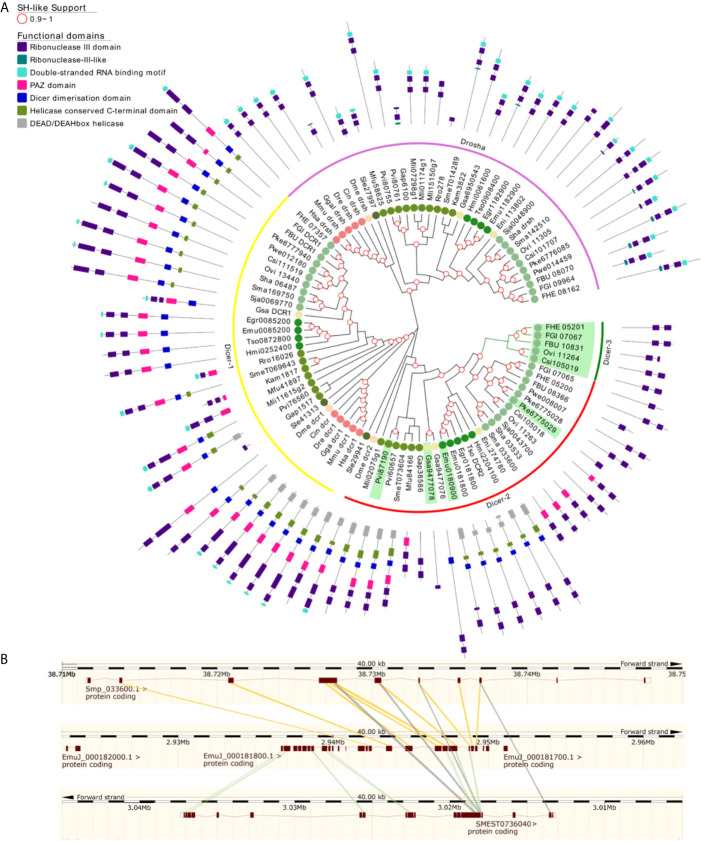
Ribonuclease III subfamilies of flatworms **(A)** A maximum likelihood tree of Ribonuclease III subfamilies. SH-like approximate likelihood ratios are indicated. Conserved protein domains were predicted with HMMScan. Dcr -2 (red arch) duplications are highlighted in green. Dcr-2 is ancestrally duplicated in FBT (dicer-3, green arch). Dcr-2 has missing domains in trematodes compared to cestodes, similar structural heterogeneity was observed between ‘turbellarians’. Abbreviations are as indicated in **Figure 2**. **(B)** High scoring pairs between Dcr-2 genes of *S. mansoni*, *E. multilocularis* and *S. mediterranea*.

Ribonuclease III family proteins represent central player in the small RNA pathways. Dicers belong to the ribonuclease III family with the ability to process dsRNA. Dicer (Dcr) is responsible for recognizing a hairpin (in pre-miRNA) or long dsRNA and processing them into 22-23 nt miRNA-miRNA* or siRNA duplexes (Jaronczyk et al., 2005). These small RNA duplexes are bound and processed by Ago proteins to form the RNA interference silencing complex (RISC). Like arthropods, flatworms have two Dcr genes, Dcr-1 and Dcr-2 (including a putative *S. leucops* Dcr-2 placed in the root of Dcr-1 group) with a paralogue of Dcr-2 in some species we named Dcr-3 (**Figure 6A**) (Gao et al., 2014; Fontenla et al., 2017).

In Neodermatans, Dcr-2 is variable in number and organization (Fontenla et al., 2017). Strikingly, trematodes in contrast to cestodes have a shorter version of Dcr-2 with only the RNAse III domains and, in some cases, a PAZ domain (**Figure 6A**). Additionally, a duplication of Dcr-2 is evident in foodborne trematodes (FBT) compared to blood flukes (BF) (**Figure 6A** green arch). In FBT Dcr-2 and Dcr-3 genes are organized as inverted tandem repeats with the exception of *F. buski* and *Paragonimus spp*, where the fragmented nature of the assembled genomes does not allow to confirm or discard this gene arrangement.

The comparison of Dcr-2 genes from the cestode *E. multilocularis*, the planaria *S. mediterranea* and the trematode *S. mansoni* showed that the second exon of Sma033600 matches with exon 15 of the cestode or planarian counterparts. In contrast, exon 1 of cestode Dcr-2 matches with exon 1 in the planarian gene (**Figure 6B**). This observation suggests that the shortening of Dcr-2 is the result of a genomic reorganization that occurred ancestrally in trematodes, probably by an unequal crossover between different chromatids or an intra-chromatid recombination that resulted in the deletion of about half of the ancestral gene. Consequently, this may have led to the absence of the helicase and dsRNA binding domains. It is tempting to speculate that these proteins might only recognize ssRNA as substrates, but experimental evidence is missing.

Although some variation in the structure of ‘turbellarians’ Dicers was also detected, we cannot rule out these may be artefactual due to the fragmented nature of these transcriptomes.

### Belle/PL10 Is Duplicated in Flatworms While Vasa Is Lost in Neodermatan Parasites

**Figure 7:**
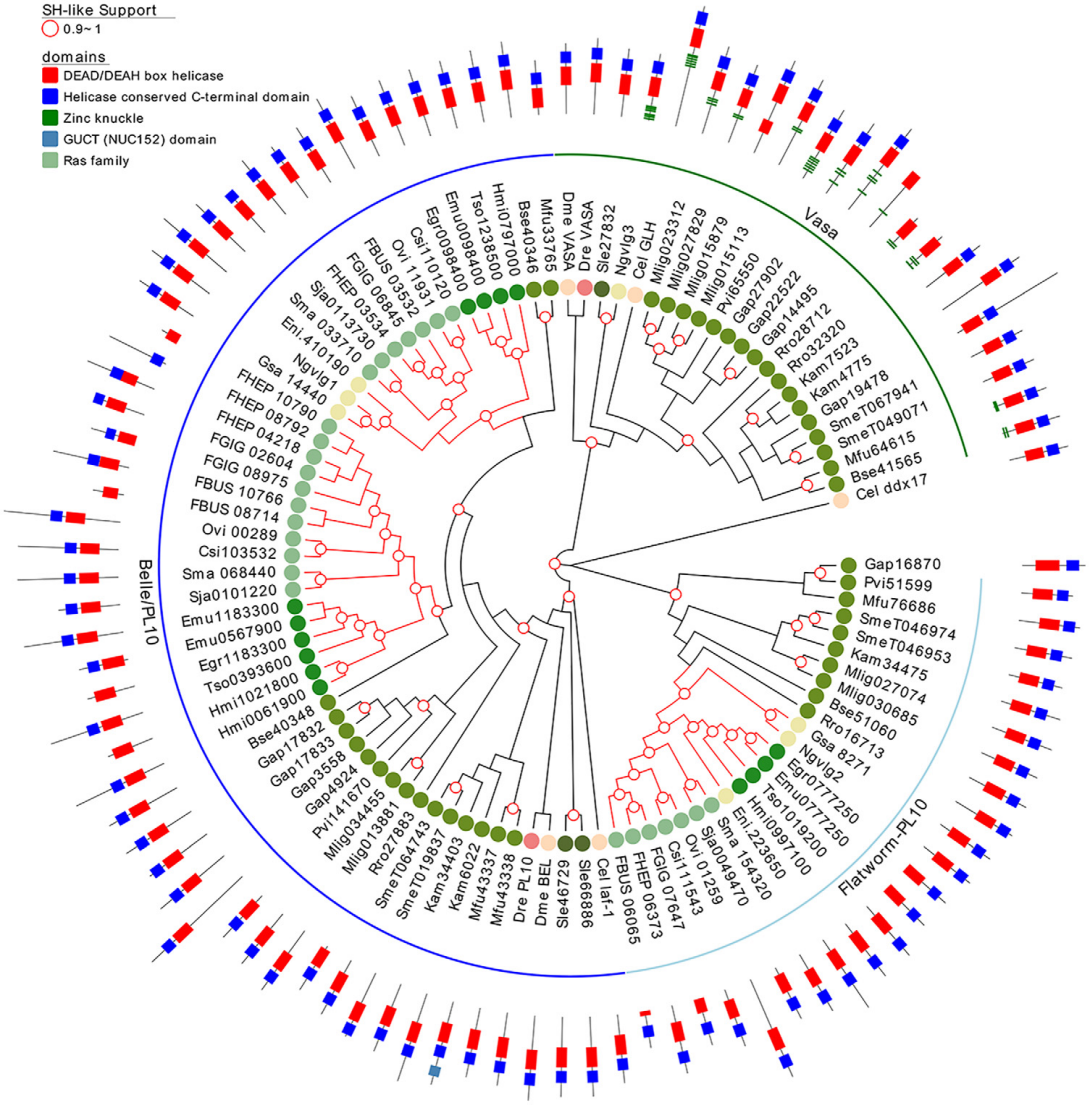
A maximum likelihood tree of Vasa and Vasa-like proteins. SH-like approximate likelihood ratios are indicated. Conserved protein domains were predicted with HMMScan. Green and blue arches correspond to Vasa and Belle/PL10, respectively. Light blue arch corresponds to flatworm PL10. Red branches correspond to Neodermatan species. Species abbreviations are indicated as in **Figure 2**.

Since several other piRNA pathway genes, besides piwi itself, seem to be absent in neodermatans, we investigated in more detail other relevant members involved in the pathway. Vasa is a germline specific DEAD box RNA helicase and plays an essential role in regulating germ cell differentiation (Abdelhaleem, 2005). Taking advantage of our extended set of transcriptomes and genomes of free-living and parasitic flatworms, we analyzed the conservation of Vasa and its paralogous gene, Belle. We founded that while ‘turbellarians’ conserved homologue genes to Vasa and Belle (green and blue arches in **Figure 7**), Neodermatans have lost vasa orthologues while maintaining Belle/PL10 homologs (blue branches in **Figure 7**). The only exception is Ngvlg3 a Vasa orthologue detected in the monogenean *Neobenedenia girellae.* Interestingly, this gene was not found to be expressed in any tissue and its knockdown by RNAi produced no phenotypic effect on the worm (Ohashi et al., 2007). Thus, we speculate that given the basal position of *N. girellae* within the Neodermatans is possible that Ngvlg3 may be a non-functional pseudogene of the free-living ancestors.

Notably, Belle-like gene is duplicated in all Neodermatans and in *B. semperi*, the closest free-living ancestor of the Neodermatans (blue arch in **Figure 7**). We also found a third group of helicases that we classified as Belle related (light blue arch in **Figure 7**) given that they show a higher percentage of identity with Belle and/or laf-1 compared to other of the *D. melanogaster*/*C. elegans* helicases (**Table S9**). All genes considered within this family have a similar domain structure, with conserved DEAD/DEAH-box and C-terminal helicase domains (**Figure 7**). An amino terminal repeat of Zinc knuckle was detected in several Vasa genes and in the *C. elegans* ortholog (GLH gene). Remarkably, Vasa homologous genes were amplified in several ‘turbellaria’ including *M. lignano* where 4 genes were detected after clustering highly similar sequences.

### Other PiwiRNA Pathway Proteins Are Conserved Only in Free Living Flatworms

**Figure 8:**
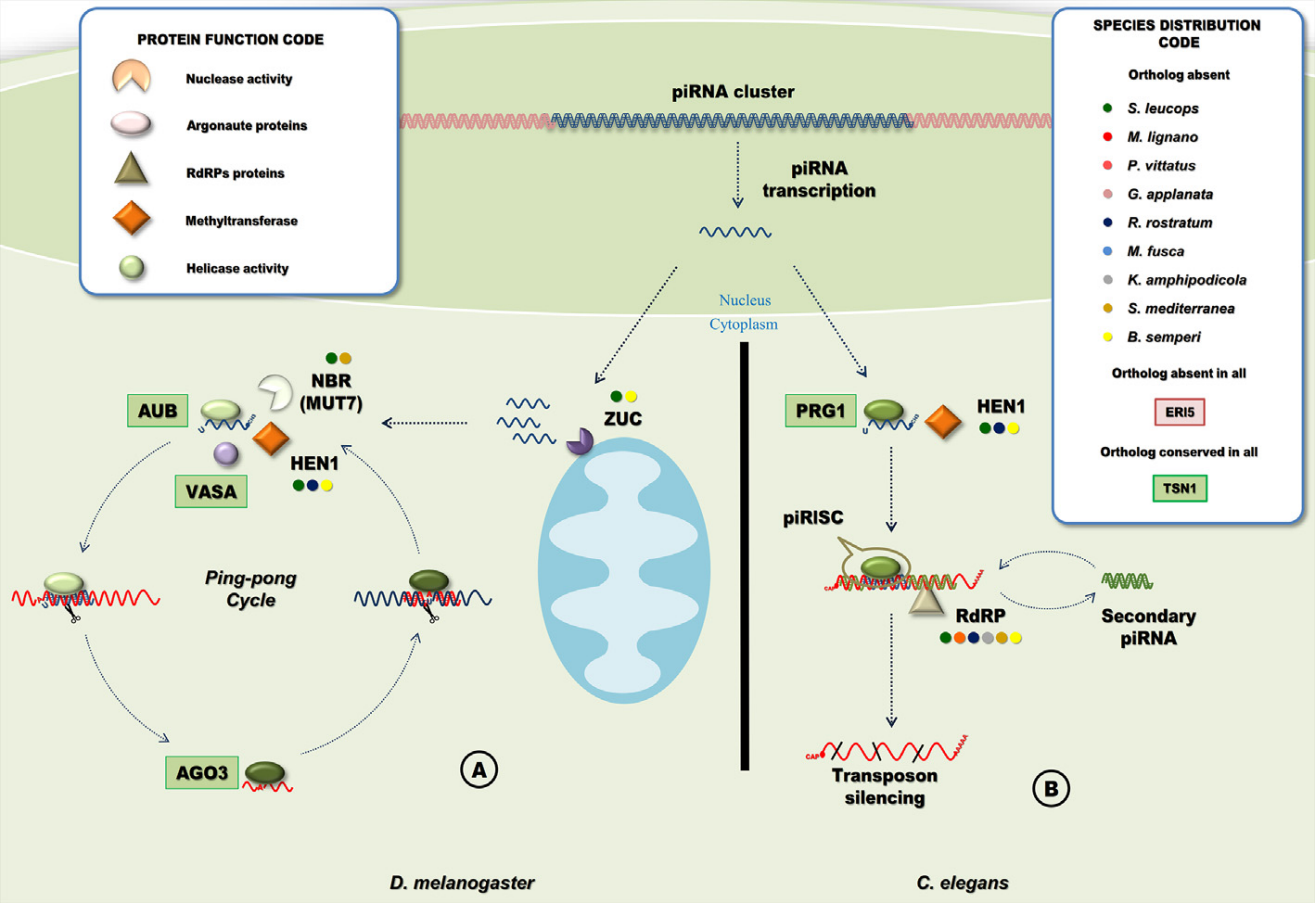
Piwi-interacting RNA pathways. **(A)** PiRNA pathway in *D. melanogaster*. PiRNA precursors are cleaved by a protein of the mitochondrial membrane, Zucchini (Zuc) producing the primary piRNAs that are loaded into Aub, maturation of piRNA requires the 2-O methylation and cleavage of the piRNA 3’ end by HEN1 and NBR (MUT7), respectively. The primary piRNA and Aub form the piRISC, secondary piRNAs are generated in a slicer-dependent amplification loop that silence cytoplasmic TE transcripts named “Ping-Pong” cycle (Tóth et al., 2016). **(B)** PiRNA pathway in *C. elegans*. In the cytoplasm, piRNAs are bounded by a PRG protein and methylated at the 3’ end by HEN1. The processed piRNA with PRG form the piRISC that will cleavage target RNA, target recognition is followed by the generation of secondary siRNAs mediated by RdRPs (Weick and Miska, 2014). Circles indicate species with missing homologous genes (“Species Distribution Code” box). A ‘shape’ code was used to indicate predicted function of factors (“Protein Function Code” box).

An RNA dependent RNA polymerase (RdRP) amplifies the signal leading to the generation of secondary siRNAs in *C. elegans*. Two ‘turbellarian’ species (*G. applanata* and *M. fusca*) had sequences with RdRPs functional domains (**Figures 8B**, **S5** and **Table S6**) in addition to *M. lignano*, (Fontenla et al., 2017). RdRPs were not detected in *S. leucops* or *P. vittatus* the two other most ancestral species of Platyhelminthes analyzed here. Interestingly, we found RdRPs in the phylum Gastrotricha, sister phylum to all Platyhelminthes (Egger et al., 2015; Laumer et al., 2015) (**Figure S5B**), suggesting that RdRPs were conserved in the common ancestor to both phyla and were lost during the evolution of Platyhelminthes. While, it is clear that the piRNA pathway does not depend on RdRPs in *S. mediterreanea* and probably other species where RdRPs are not present, it might be possible to occur in species where RdRPs are conserved, as in *C. elegans* (**Figure 8B**).

We also found homologues to other piwi pathway genes like HEN1, Zuc or Mut7 in almost all ‘turbellarian’ species although they are completely absent in neodermatans. (**Figures 1**, **8A** and **Table S6**). MUT-7 is a protein that contains an exonuclease domain that, in complex with RDE-2, is required in the RNAi pathway in *C. elegans* (Tops et al., 2005)(**Figure S4**). However, in *D. melanogaster* RDE-2 is missing and the orthologue of MUT-7, Nibbler (Nbr), is involved in the maturation of piRNA 3’ ends (Wang et al., 2016). As in insects, RDE-2 is missing in flatworms suggesting that the MUT-7/Nbr homolog in ‘turbellarians’ is involved in piRNA biogenesis but not in the RNAi pathway in free living flatworms (**Figures 1**, **8A** and **Table S6**).

## Discussion

Platyhelminthes comprise one of the early diverging phyla of bilateral metazoans, originated approximately 839 million years ago (Hedges et al., 2015), inhabiting a wide range of ecosystems and particularly successful in adapting to parasitic life. Since small non-coding RNAs have been implicated in regulating developmental transitions, we reasoned that they might be involved in the adaptation to parasitism way of life. Therefore, comparing the conservation of small RNA pathways among the paraphyletic group of free-living (‘turbellarians’) and the monophyletic parasitic Neodermatans may provide evolutionary clues to disentangle possible adaptations to parasitism.

### The Argonaute Gene Family

We have previously shown (Fontenla et al., 2017) that key proteins involved in small RNA pathways are conserved in all major clades of Platyhelminthes; however, clear differences between clades are evident, particularly the complete absence of the Piwi pathway genes in Neodermatans.

While miRNA class Ago proteins are conserved in all flatworms, a platyhelminth-specific family that we named FLAgos (Fontenla et al., 2017), showed independent gene amplifications in Trematodes and Cestodes but not in ‘turbellarians’, with the only exception of a duplication in *S. mediterranea* (**Figure 3**). This group might have originated as a highly divergent siRNA class Ago, that was further amplified and diversified. On the other hand, while the parasitic Neodermatans lack Piwi-like proteins, these are amplified in free-living flatworms, including the canonical Piwi 2 class, and a new group, the FLiwis, specific of free living Rhabditophora (**Figure 4**). As in FLAgos, since Piwi1 class homologues are missing, is possible that the Fliwi group represents a fast-evolving piwi1 class that diverged within flatworms. Interestingly, neither FLAgos, nor FLiwi are shared by other lophotrochozoans lineages analyzed in this study, despite amplifications can also been detected within them.

Piwi proteins are involved in the biogenesis and activities of piRNAs, being crucial at silencing transposable elements. In *S. mediterranea*, Piwi genes were reported to be essential in the regeneration and homeostasis of neoblasts, the pluripotent stem cells of Platyhelminthes (Reddien et al., 2005; Palakodeti et al., 2008). Additionally, *S. mediterranea* piwi-1 (SmeT036375) is highly expressed in blastomeres and is critical during embryogenesis and organogenesis (Davies et al., 2017). The highest levels of piwi-1 are found in epidermal progenitors and tetraspanin-1-positive neoblasts. Differentiation of pluripotent neoblasts into fate-determined progenitors and terminally differentiated cells is accompanied by a successive reduction of piwi-1 levels (Kim et al., 2020). In *M. lignano*, piwi-1 (Mli034222) but not piwi-2 (Mli016226) was found to be involved in the piRNA pathway in both germline and somatic cells, as well as in the maintenance of stem cells (Zhou et al., 2015). It is of interest to decipher if similar functional restrictions or labor division is found in other ‘turbellarians’, now that we show that the key genes are conserved.

FLAgo proteins show less sequence conservation, due to a higher substitution rate, a feature generally associated with acquiring novel functions. We show evidence of site-specific diversifying selection mainly in FLAgos compared to other Argonaute groups. We hypothesize that the sequence variation observed for FLAgos might have been associated to specialization in the gene function within this cluster. Further experimental evidence is needed in to validate this hypothesis, for instance evaluating if the substitutions detected are associated with changes in functional domains or protein conformations. Even more, flatworm-specific proteins like the FLAgos (not conserved in the host) could be targets for new drugs or vaccines. In that sense, a drug that specifically target this highly diverting subgroup could be a potential innovation in the treatment of helminthiasis. Such drug has already been proposed in *in silico* modeling to target Hsa Ago-2 (Schmidt et al., 2013).

### The Dicer Family

In flatworms, the Dicer family is organized in two subgroups. While the Dcr-1 group is invariable with only a single gene per species, the Dcr-2 group is heterogenous both in number of copies and structure (**Figure 6A**). The shortening of Dcr-2 is probably the consequence of a genomic reorganization that eliminated the first 14 exons of the gene in the ancestor of all trematodes (**Figure 6B**). Transposable elements (TE) are recognized as contributors to genomic innovation as well as genomic instability across a wide variety of species (Klein and O’Neill, 2018). We have previously reported the accumulation of TEs in the genomes of trematodes, especially FBT, as an extreme example, more than 50% of the genomes of *Fasciola* spp. corresponds to repetitive elements (Choi et al., 2020). It is tempting to speculate that the accumulation of TEs in the ancestor of the trematode class has driven the Dcr2 reorganization and, possibly, further accumulation of TEs in the FBTs contributed to generate a duplication of Dcr2 (Dcr3) in that linage. Whether these shorter Dcr 2/3 are functional or represent a pseudogene awaits confirmation. However, while Dcr1 and Dcr2 are express across different stages, Dcr3 seems to be limited to eggs (Fontenla et al., 2017).

The detection of Dcr-2 proteins in *G. applanate* and *M. fusca* with very similar structures to the ones in trematodes, may indicate that the genomic reorganization detected in trematodes may have occurred more than once during the evolution of flatworms. However, given the fragmentation of the ‘turbellarian’ transcriptomes, further genomic data are needed to verify these observations.

### The Vasa and Vasa-Like Genes

Vasa genes in planarians are expressed in ovary and testis of sexual worms and in the totipotent cells (neoblasts) of asexual ones (Shibata et al., 1999). Increase in the expression of Vasa was detected in growing blastema of regenerating planarians and lost in irradiated organisms (Shibata et al., 1999). Belle (also known as PL10), on the other hand, is a Vasa-related protein, that has conserved roles in fertility and development, and co-localizes with Vasa to the germline (Johnstone et al., 2005). Vasa was proposed to be part of the biogenesis of piRNAs and to be differentially conserved between ‘turbellarians’ and Neodermatans (Skinner et al., 2014). Like the absence of Piwi, the absence of Vasa in Neodermatans results puzzling. Piwi and Vasa are widely known among developmental biologists as germline markers. Vasa was even found to be expressed in the germline of early branching Metazoans like the ctenophore clade, suggesting a central role in the development of all Metazoans (Mochizuki et al., 2001; Rebscher et al., 2007; Alié et al., 2011). Even more, vasa mutants in *D. melanogaster* fail to form pole cells, the precursor of the gonadal germ cell population, and show deletions of abdominal segments (Schupbach and Wieschaus, 1986). It has been proposed that Vasa genes arose by duplication of an ancestral PL-10-related gene before appearance of sponges but after the diversion of fungi and plants (Mochizuki et al., 2001). Our data suggest that a second duplication of PL-10 took place in an early ancestor of the Platyhelminthes producing a flatworm specific family of PL-10-related genes. If that is the case, we speculate that a redundant role of Vasa, PL-10-related and flatworm PL-10 in ‘turbellarians’ was further simplified in Neodermatans with the loss of Vasa. Why the loss of Vasa was evolutionary favored in Neodermatans is still unknown; however, the germline expression and role in the gametogenesis of flatworm specific PL-10 has been reported in Neodermatans by RNAi assays (Ohashi et al., 2007). Moreover, vasa-like genes, i.e. PL-10 are strongly expressed in the ovary of *Schistosoma mansoni* female adult worms and showed high expression in female adults and eggs laid *in vitro* by worms in culture (Skinner et al., 2012). More recently, RNAi against vasa/PL10-like gene -1 in *S. mansoni* adult female worms resulted in smaller ovaries and a reduced number of ovarian dividing cells (Skinner et al., 2020). Similarly, the knockdown of vasa-like genes in *S. japonicum* induced changes in the morphology of the reproductive organs, especially in the female ovary, vitellarium and the male testes. In addition, a significant reduction in egg production in knocked-down parasites was evident (He et al., 2018).

### PiRNA Biogenesis in ‘Turbellarians’

The piwiRNAs (piRNAs) are generated either from RNA transcripts of active transposable element (TE) copies or from transcripts originated from specialized loci in the genome called piRNA clusters. In general, piRNAs generated from piRNA clusters are mostly antisense to TE mRNA sequences (Tóth et al., 2016). However, these regulatory non-coding RNAs are also originated from different biogenesis pathways depending on the species. In *D. melanogaster* and vertebrates, piRNAs are 26-30 nt in length. These are derived from single-stranded piRNA precursors and processed in the cytoplasm by Zucchini (Zuc). This is a protein with endonuclease activity for single-stranded RNA and expressed in the mitochondrial surface with a predicted phospholipase D-like domain (**Figure 8A**). The piRNAs generated this way preferentially have a 5’-end uracil. HEN1 is required for 2’-O-methylation of maturing piRNAs (Horwich et al., 2007; Montgomery et al., 2012). Mature piRNAs are bounded by the Piwi protein Aubergine (Aub), to form the piRISC that targets and degrades TE mRNAs. Ago3, on the other hand, binds to TE mRNA cleaved sequences that contains an adenosine residue at position 10 and will target piRNA sequences resulting in an amplification loop named ping-pong cycle (Weick and Miska, 2014). The ping-pong cycle includes not only Aub and Ago3, but also Vasa, that has two proposed roles in piRNA processing. First, Vasa participates in the assembly of the ping-pong complex (Xiol et al., 2014). Second, the RNA-unwinding activity of Vasa helps to release cleaved products from the piRNA-protein complex to facilitate the ping-pong cycle (Nishida et al., 2015).

*C. elegans* piRNAs are shorter (21 nt long), also with a 5’ uracil, and require methylation by HEN1 for maturation. Mature piRNAs bound to the Piwi orthologue PRG1, form the piRISC complex that targets and silence TE mRNAs. However, instead of the ping pong mechanism, the amplification of the silencing signal relies on RNA dependent RNA polymerases (RdRPs)(**Figure 8B**) (Weick and Miska, 2014).

The conservation of Zuc, Nbr, Vasa and RdRPs in some ‘turbellarian’ species rises a question about the biogenesis of piRNAs in the free-living clades. In *M. lignano*, it has been reported that the knock down of vasa produce a severe reduction in the piRNA fraction (Zhou et al., 2015). Additionally, in *M. lignano*, *S. mediterranea* and *Dugesia japonica* where the small RNA population has been sequenced, piRNAs are ~32 nt in length preferentially displaying U at the 5’ end (Palakodeti et al., 2008; Friedländer et al., 2009; Qin et al., 2012; Zhou et al., 2015) like the ones described in *D. melanogaster*. Besides, a preference for A at position 10 and the overlap of reads by 10 nt suggest that a ping-pong cycle occur in these species with no evidence of any other mechanism of amplification. Therefore, it is possible to speculate that the RdRPs detected in *M. lignano* (and some other ‘turbellarians’) might not be involved primarily in the amplification of piRNAs. In any event, additional experimental evidence, possibly involving RNAi against RdRPs genes and sequencing of the small RNA population, is needed to test this hypothesis.

### Alternative Solutions to Piwi Absence in Parasites

The absence of Piwi in addition to the amplification of the FLAgos in Neodermatans raise the hypothesis that some of the FLAgos substitute the role of the Piwi proteins in this clade (Skinner et al., 2014). In this regard, Cai et al. (2012) sequenced the population of small RNAs associated to the FL-Ago SjAgo2 (Sja_0045200) and found that it was preferentially associated with siRNAs derived from LINE and LTR retrotransposons, the main targets of Piwi proteins (see below). This observation suggests that the FLAgos could be at least partially mimicking the role of the lost Piwi genes in Neodermatans. Furthermore, the silencing of SmAgo2 resulted in a moderate increased expression of transposable elements, suggesting that this protein might be involved in regulating transposons in *Schistosoma mansoni* (Protasio et al., 2020).

The absence of piwi pathway proteins in parasitic species seems to be a consistent trend. We here provide strong evidence of the complete absence of the pathway across all parasitic flatworms. Similarly, the absence of Piwi have been reported in all nematode clades, except clade V, the one containing the model species *C. elegans*, and some animal parasites as *Haemonchus contortus* and *Pristionchus pacificus* (Sarkies et al., 2015). Other proteins of the piwi pathway were also absent in non-clade V nematode, confirming the absence of a functional pathway. Furthermore, the piwi pathway was also found absent in the dust and scabies mite genomes (Mondal et al., 2018). Both in nematodes and in dust mites there is evidence that alternative siRNA related mechanisms are involved in controlling TEs. The amplification of genes associated with the main small RNA pathways in parasitic flatworms is suggestive of a similar cooption of functions. Furthermore, as already mentioned primary evidence show that this might be the case in parasitic flatworms (Cai et al., 2012; Protasio et al., 2020). Since TEs are recognized as contributors to genomic innovation as well as genomic instability across a wide variety of species (Klein and O’Neill, 2018), is tempting to speculate that piwi loss might be associated to rapid genomic reorganization leading to adopt a parasitic way of life.

## Conclusions

We provide a strong bioinformatic support for the presence and absence of key proteins involved in small RNA pathways in early diverging free-living flatworms, suggesting that miRNA regulation, piRNA mediated silencing and RNAi are ancestral regulatory mechanisms in flatworms. In addition, differences observed in later evolving parasitic species strongly suggest that small RNA mediated mechanisms might have been also relevant during the transition to parasitism.

A long and still unsettled discussion has taken place regarding the biological simplification occurred in flatworms, especially in the parasitic Neodermatans. It is unclear, yet, if the process of loss of redundancy is the product of an adaptive mechanism to parasitism (Tsai et al., 2013; McNulty et al., 2017) or is an ancestral characteristic acquired by the Neodermata clade (Hahn et al., 2014). Interestingly, in the present study we found that the ‘turbellarian’ parasite *K. amphipodicola* showed no major differences in the conservation of small RNA pathway factors respect to other phylogenetically related free-living ‘turbellarians’, like *S. mediterranea*, including the conservation of a putative functional piRNA pathway that has been lost in Neodermatans (Skinner et al., 2014). Then, it is possible to hypothesize that the overall simplification associated to the Neodermatans is not a characteristic needed for parasitism in the phylum Platyhelminthes. However, the complete absence of the piwi pathway mediators in all trematodes and cestodes is suggestive of an early loss in an ancestor of the Neodermatans. This single loss might have had dramatic evolutionary consequences, since transposable elements might have driven genome instability that led to biochemical, morphological and functional transformations, for instance the origin of the neodermis (Caira and Littlewood, 2013), and other changes that favored the adaptation to a novel lifestyle. Along these lines, it is tempting to think that the later independent amplification of FLAgos, and Belle/PL10 in trematodes and cestodes might have resulted from an adaptation to these changes, either for control of transposons, and/or generating novel regulatory mechanisms mediated by small non-coding RNAs.

Different experimental approaches can be considered to test this hypothesis. Functional genomic tools tested in flatworms like RNAi (Dell’Oca et al., 2014), transgenesis (Rinaldi et al., 2012; Suttiprapa et al., 2016), genome-editing by CRISPR-Cas9 (Lok et al., 2017; Ittiprasert et al., 2019; Sankaranarayanan et al., 2020) and immunoprecipitation assays (Free et al., 2009) could be used to define the function of the factors reported here or to detect novel ones. Additionally, experimental evidence involving chromatin immunoprecipitation of nucleosome core particles followed by high throughput sequencing has proven to be useful to detect chromatin modification triggered by dsRNA (Gu et al., 2012).

To conclude, these findings together with our previous report (Fontenla et al., 2017), describe novel features of the biology and evolution that are unique to Platyhelminthes, implying that subtle mechanisms involved in the small RNA pathways of flatworms are different to the ones described in model organisms like mammals, *C. elegans* or *D. melanogaster*.

## Data Availability Statement

The original contributions presented in the study are included in the article/**Supplementary Material**. Further inquiries can be directed to the corresponding authors.

## Author Contributions

SF performed the acquisition, bioinformatics analysis and interpretation of data and contributed in writing the manuscript. GR was involved in drafting the manuscript and critical revision of its content. JT participated in the design of the study and the interpretation of data, drafting the manuscript and critical revision of its content. All authors contributed to the article and approved the submitted version.

## Funding

The research was founded by grant Iniciación a la Investigación 2019 (CSIC-UdelaR). SF, GR and JT are researchers from the Sistema Nacional de Investigadores (SNI-ANII). JT is also researcher from Pedeciba.

## Conflict of Interest

The authors declare that the research was conducted in the absence of any commercial or financial relationships that could be construed as a potential conflict of interest.

## Footnotes

1 Here and across the manuscript we used the old term ‘turbellarian’ as a proxy to refer to all the non-Neodermatan species, including the Catenulida and the early diverging Rhabdithopora taxons.
