# Supplementary figures and images for: Lost and Found: Piwi and Argonaute Pathways in Flatworms
Source: Front Cell Infect Microbiol. 2021 May 27;11:653695. doi: 10.3389/fcimb.2021.653695 (PMC8191739; doi:10.3389/fcimb.2021.653695)

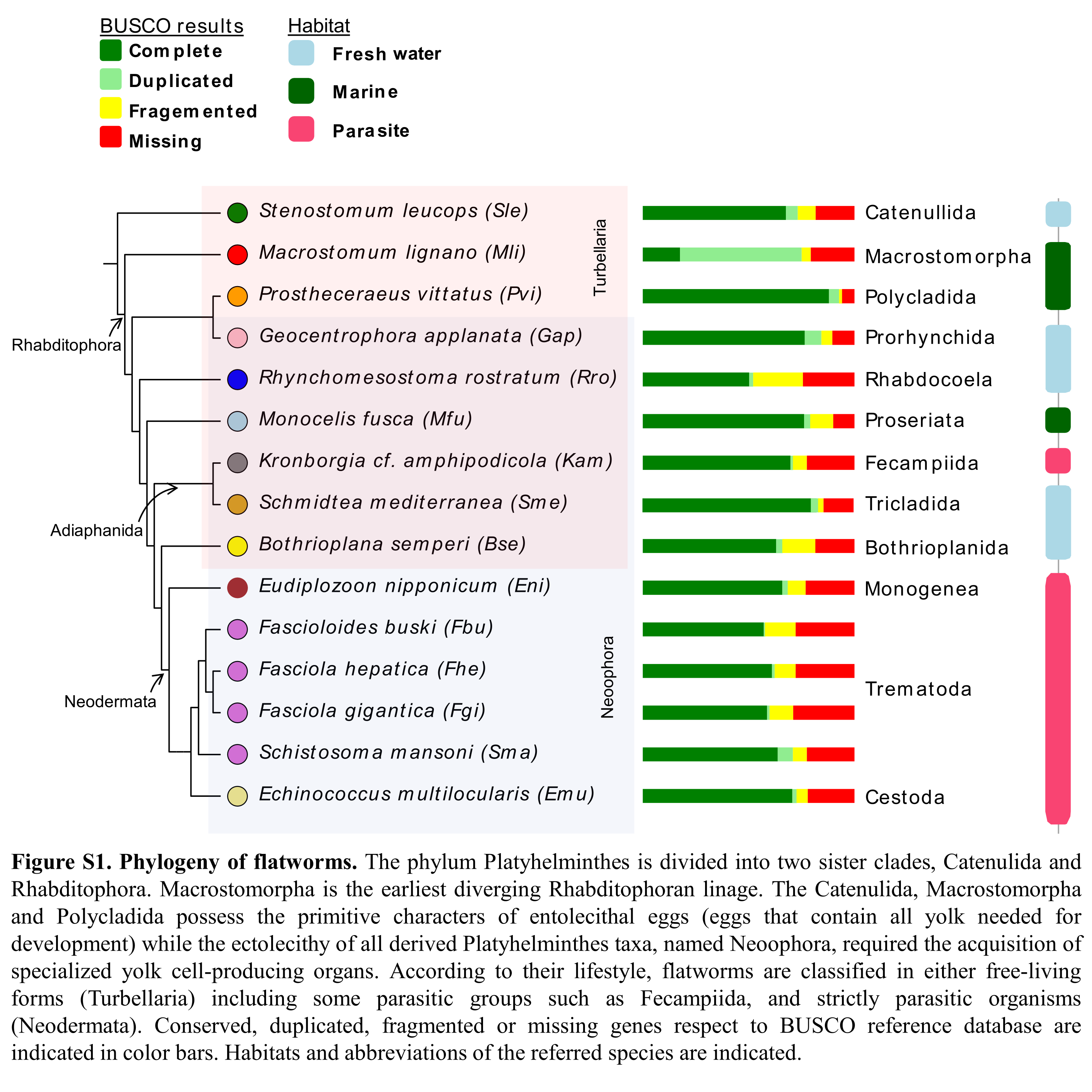

Supplement: Supplementary file 1 [file Image_1.tif]

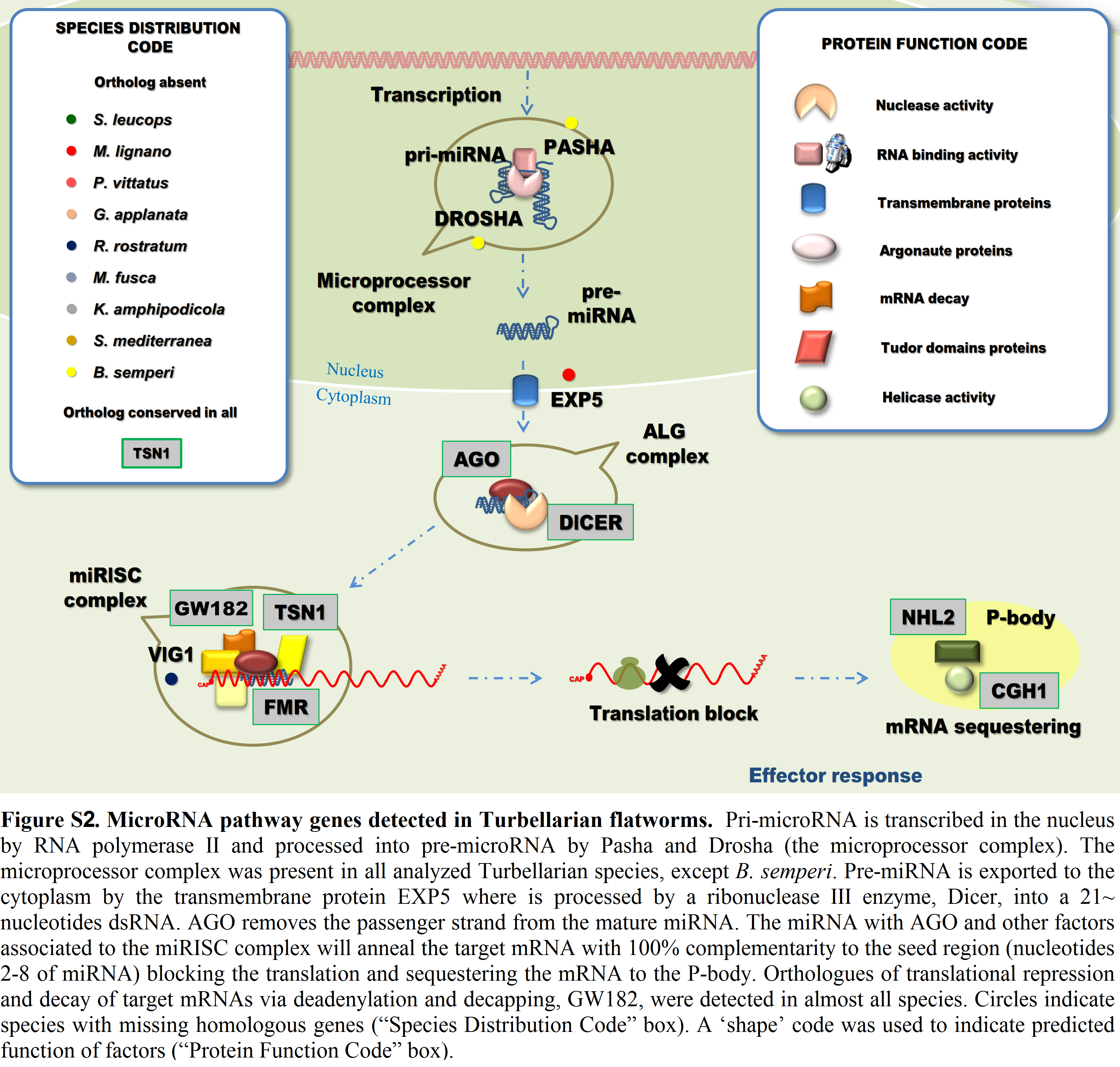

Supplement: Supplementary file 2 [file Image_2.tif]

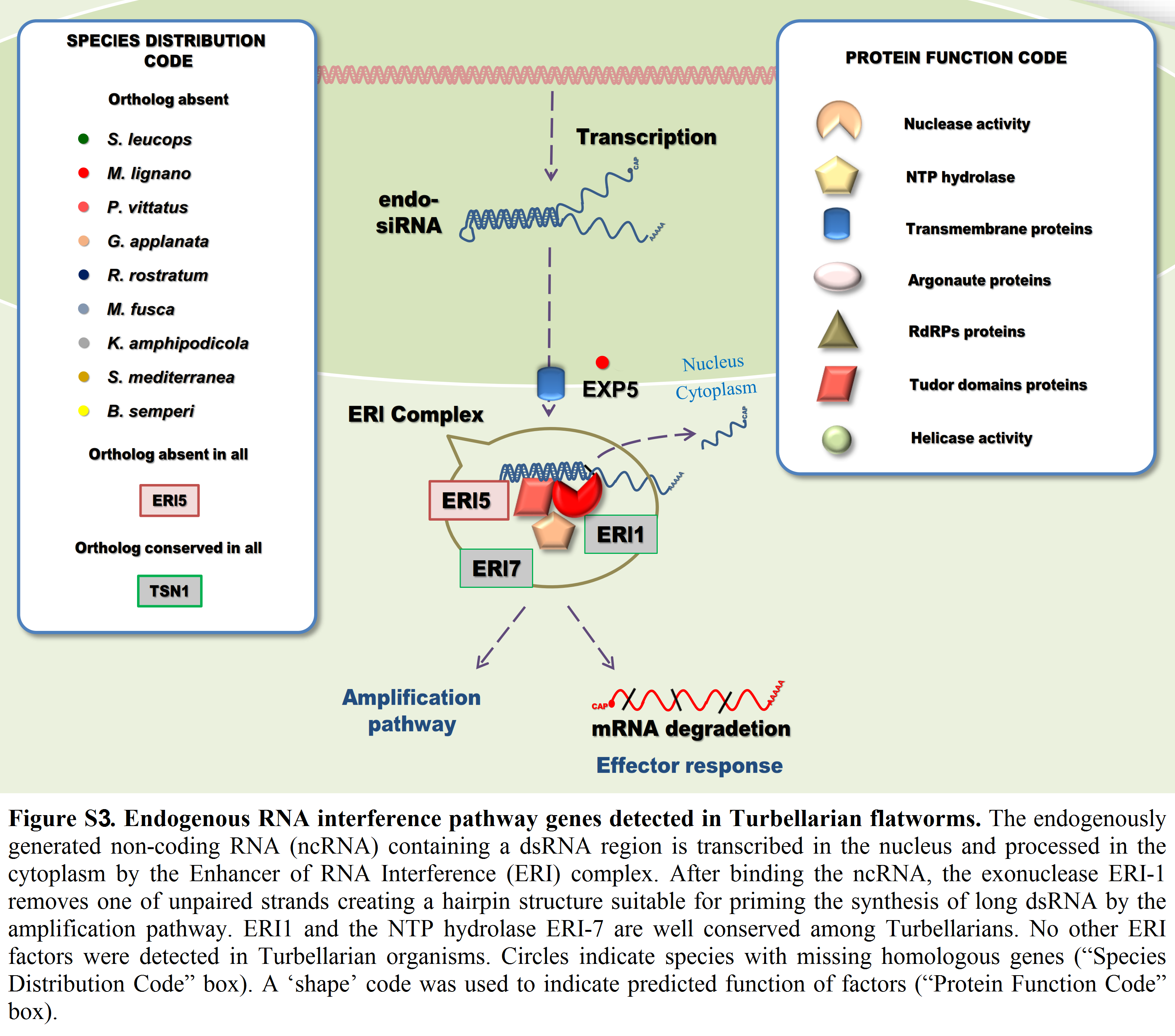

Supplement: Supplementary file 3 [file Image_3.tif]

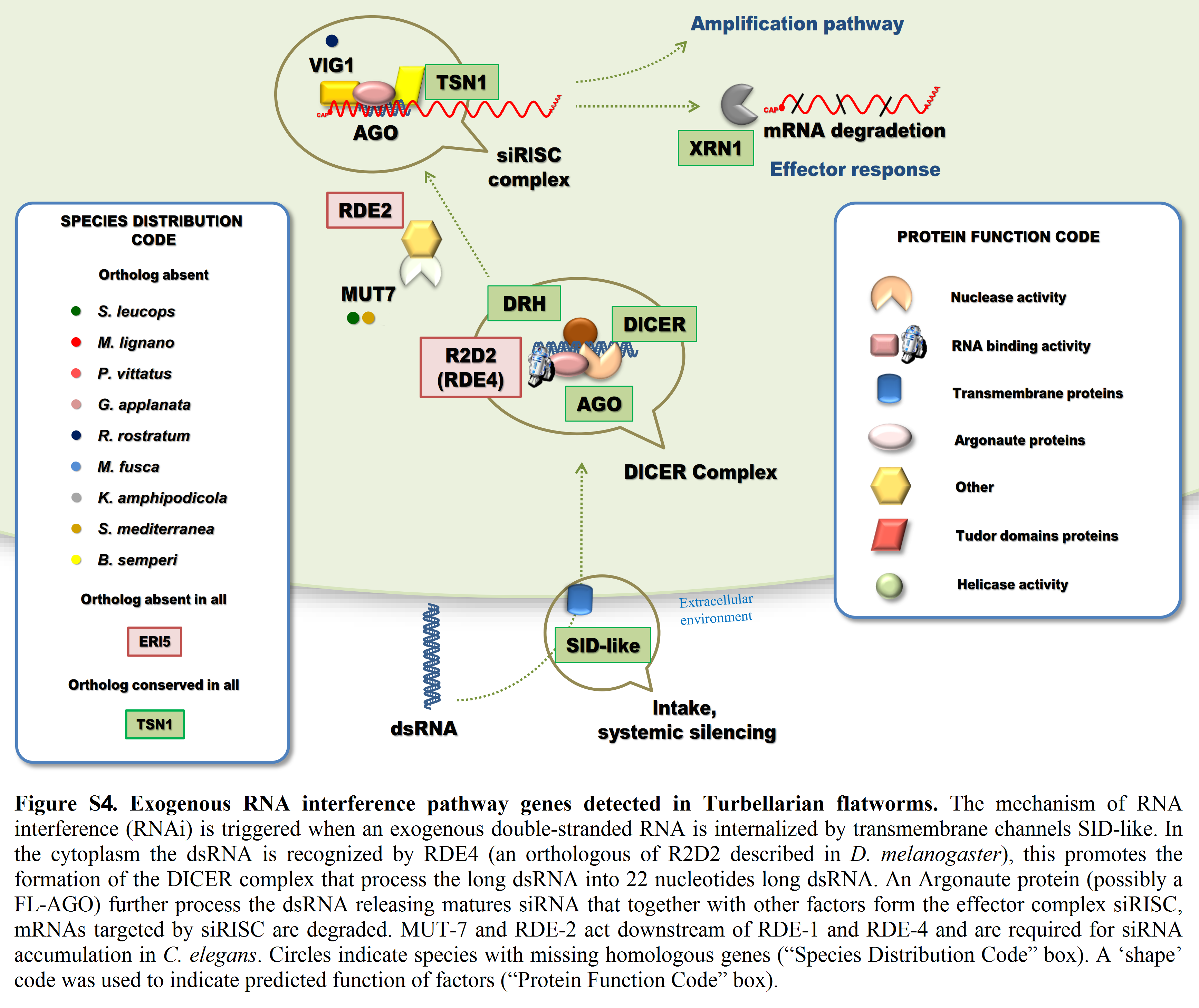

Supplement: Supplementary file 4 [file Image_4.tif]

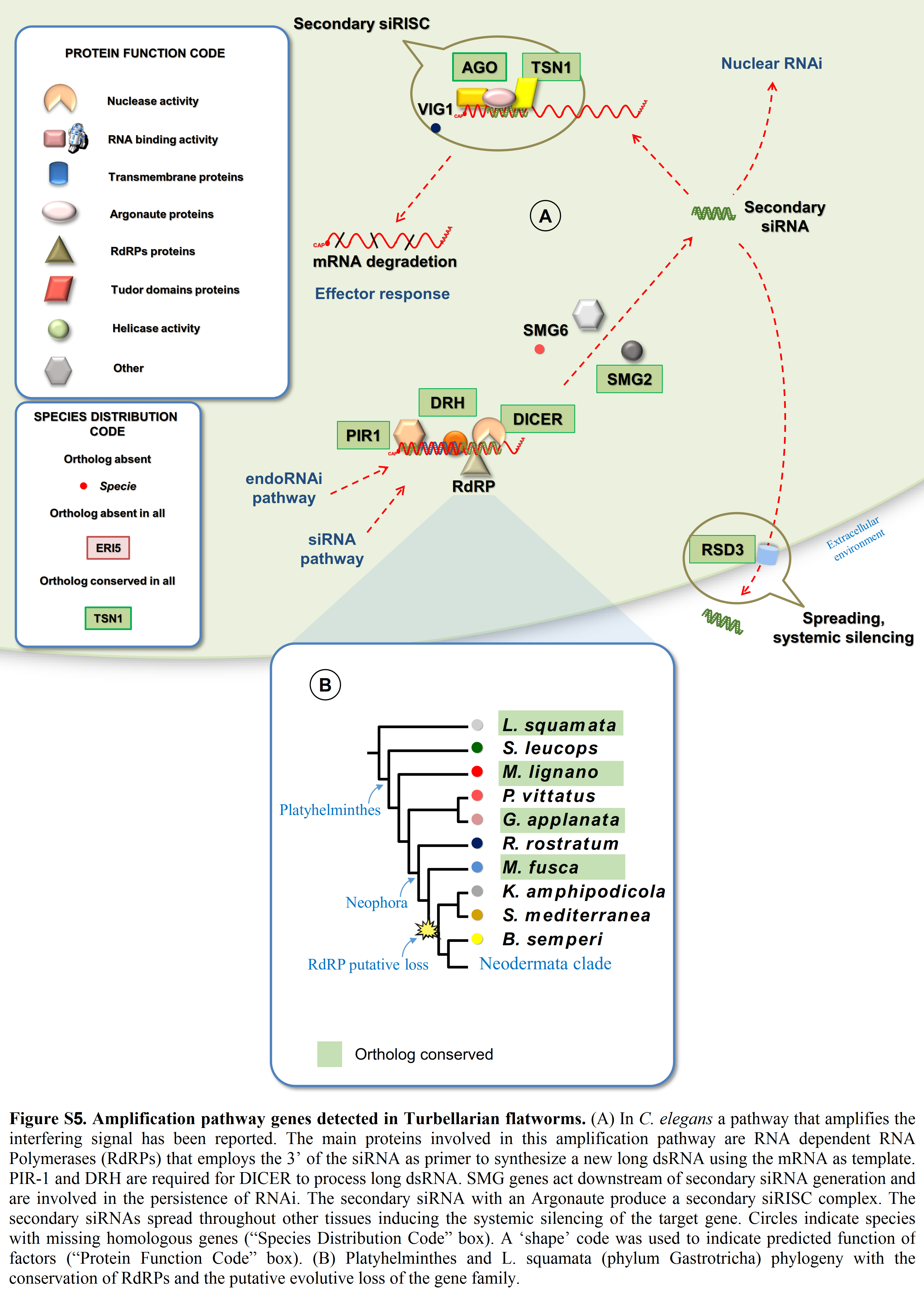

Supplement: Supplementary file 5 [file Image_5.tif]

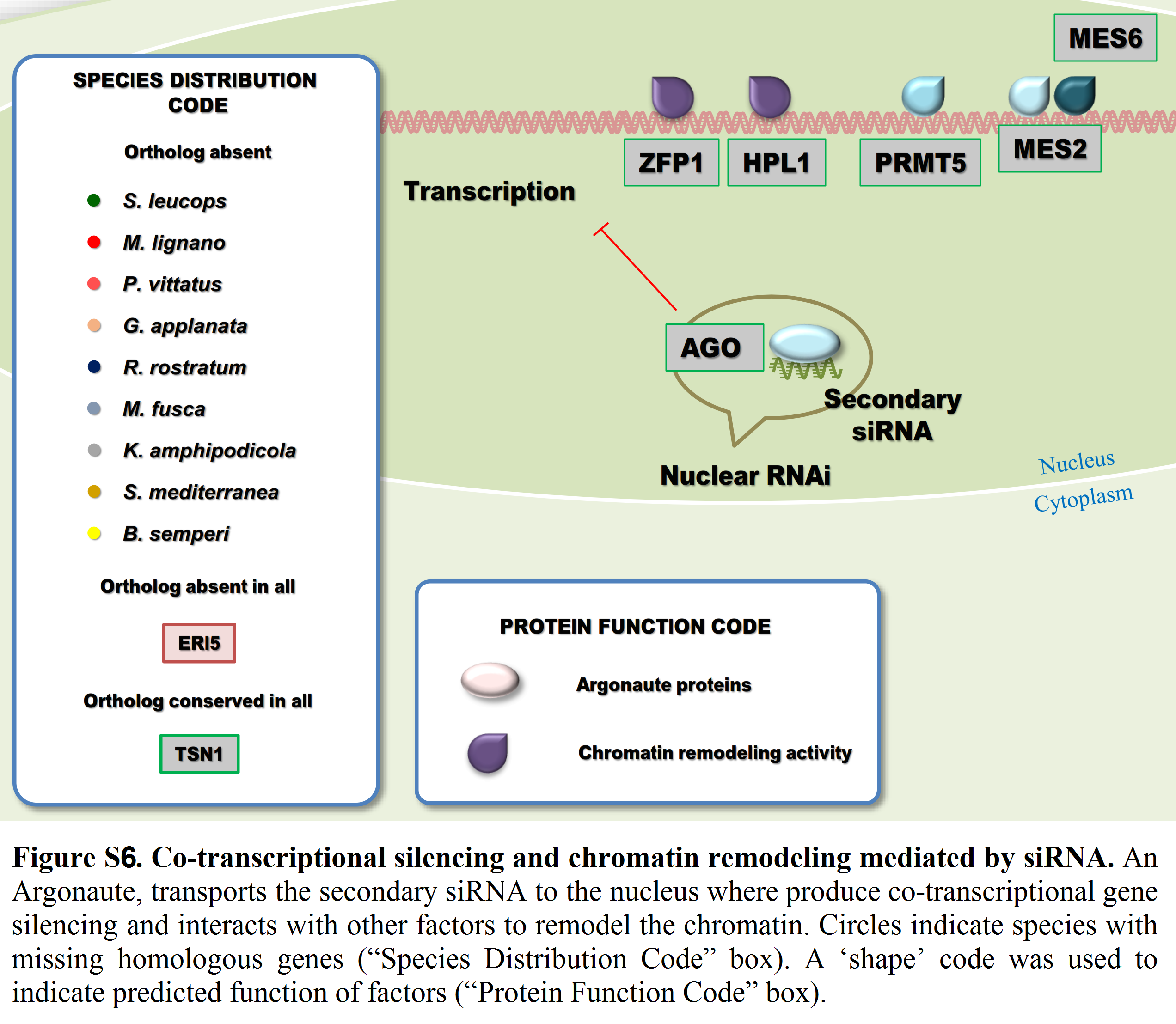

Supplement: Supplementary file 6 [file Image_6.tif]

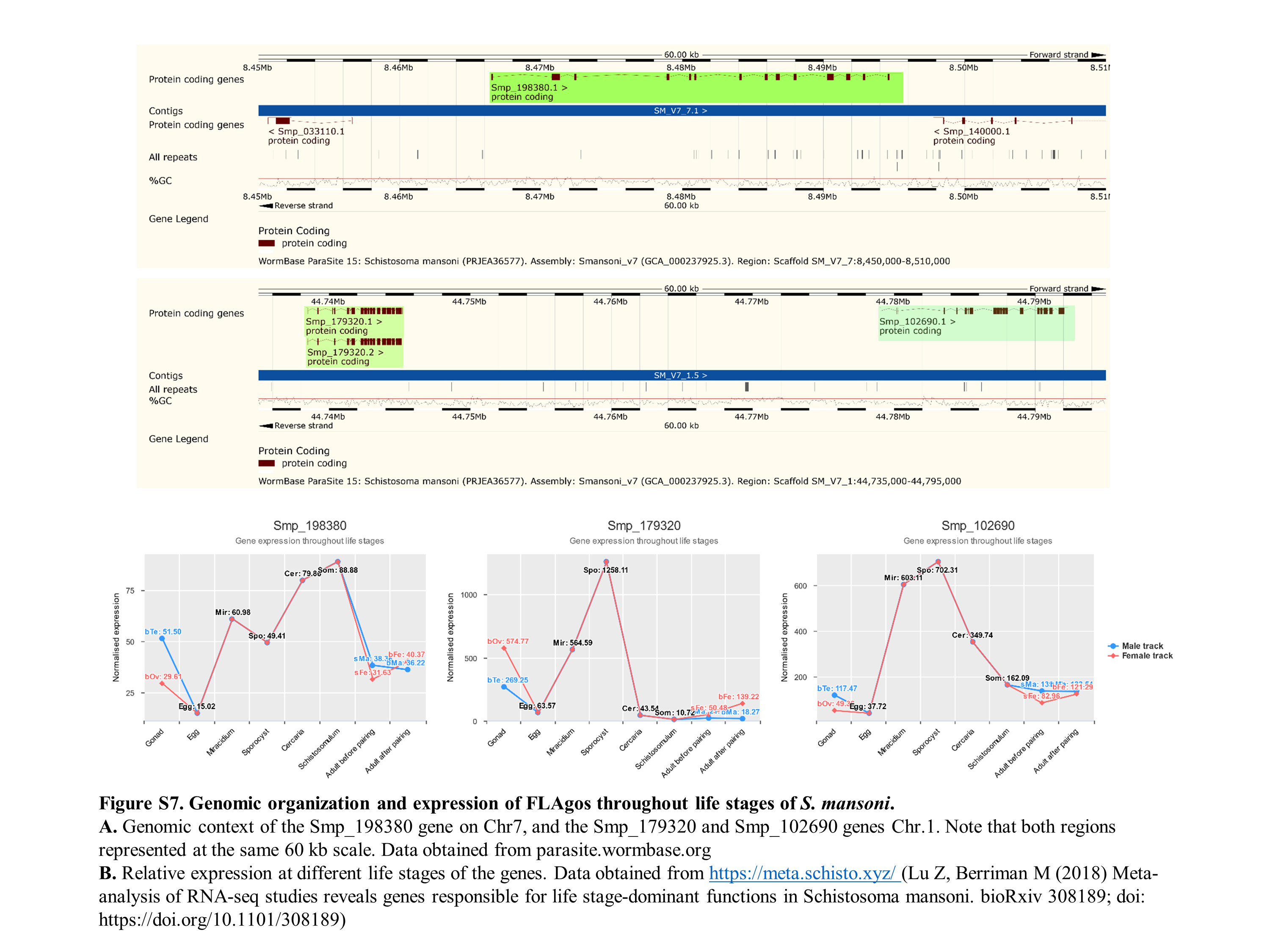

Supplement: Supplementary file 7 [file Image_7.tif]

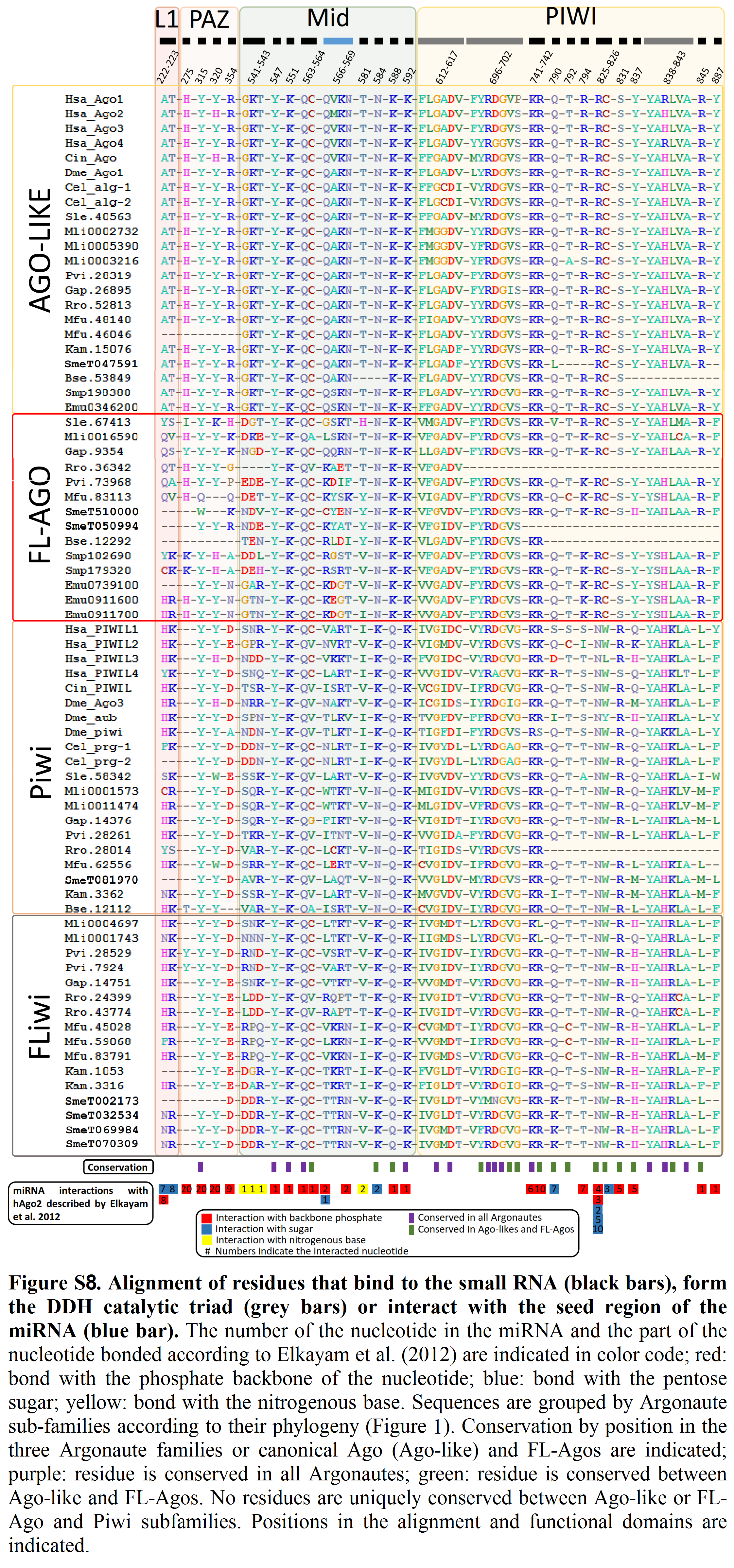

Supplement: Supplementary file 8 [file Image_8.tif]
